# Is geography an accurate predictor of evolutionary history in the millipede family Xystodesmidae?

**DOI:** 10.7717/peerj.3854

**Published:** 2017-10-12

**Authors:** Jackson C. Means, Paul E. Marek

**Affiliations:** Department of Entomology, Virginia Polytechnic Institute and State University (Virginia Tech), Blacksburg, VA, United States of America

**Keywords:** Xystodesmidae, Evolution, Gonopod, Homoplasy, *Sigmoria whiteheadi*, Diplopoda, Phylogeny

## Abstract

For the past several centuries, millipede taxonomists have used the morphology of male copulatory structures (modified legs called gonopods), which are strongly variable and suggestive of species-level differences, as a source to understand taxon relationships. Millipedes in the family Xystodesmidae are blind, dispersal-limited and have narrow habitat requirements. Therefore, geographical proximity may instead be a better predictor of evolutionary relationship than morphology, especially since gonopodal anatomy is extremely divergent and similarities may be masked by evolutionary convergence. Here we provide a phylogenetics-based test of the power of morphological versus geographical character sets for resolving phylogenetic relationships in xystodesmid millipedes. Molecular data from 90 species-group taxa in the family were included in a six-gene phylogenetic analysis to provide the basis for comparing trees generated from these alternative character sets. The molecular phylogeny was compared to topologies representing three hypotheses: (1) a prior classification formulated using morphological and geographical data, (2) hierarchical groupings derived from Euclidean geographical distance, and (3) one based solely on morphological data. Euclidean geographical distance was not found to be a better predictor of evolutionary relationship than the prior classification, the latter of which was the most similar to the molecular topology. However, all three of the alternative topologies were highly divergent (Bayes factor >10) from the molecular topology, with the tree inferred exclusively from morphology being the most divergent. The results of this analysis show that a high degree of morphological convergence from substantial gonopod shape divergence generated spurious phylogenetic relationships. These results indicate the impact that a high degree of morphological homoplasy may have had on prior treatments of the family. Using the results of our phylogenetic analysis, we make several changes to the classification of the family, including transferring the rare state-threatened species *Sigmoria whiteheadi* Shelley, 1986 to the genus *Apheloria* Chamberlin, 1921—a relationship not readily apparent based on morphology alone. We show that while gonopod differences are a premier source of taxonomic characters to diagnose species pairwise, the traits should be viewed critically as taxonomic features uniting higher levels.

## Introduction

The Appalachian Mountains have ancient origins and hold a considerable diversity of endemic species. Among the biodiversity encompassed by these mountains, wingless and low-mobility animals such as millipedes, harvestmen, snails, and salamanders are tightly coupled to their habitat. As a result of its complex topography, varied edaphic qualities, and ancient origins, the Appalachian Mountains have fostered the isolation and diversification of these low-mobility groups resulting in high species diversity in relatively small geographic areas (Marek, 2010; Hedin & McCormack, 2017). This is particularly the case for millipedes, and for the past 10–20 million years the group has had the opportunity to diversify in the Appalachian region as a result of its stable mesic environment, calcareous geology (millipedes biomineralize calcium carbonate into their cuticle), and historical contingency (Briggs, Plint & Pickerill, 1984; Shear & Selden, 1995).

The millipede family Xystodesmidae has its greatest diversity in the region, and is generally comparable in global biogeography, known species richness, and habitat preferences to the lungless salamanders of the family Plethodontidae (Mueller et al., 2004; Shen et al., 2016). However, relative to vertebrate groups, many xystodesmid taxa remain undescribed. In the case of the US genus *Nannaria*, there are 22 nominal species and ca. 60–200 are estimated in the Appalachian Mountains alone (Hoffman, 1964; Shelley & Whitehead, 1986). (Note: for taxonomic authorities of all xystodesmid millipede taxa referenced in this article see Marek, Tanabe & Sierwald, 2014.) Xystodesmid millipedes belong to the order Polydesmida, which is the most species rich order in the class Diplopoda, ca. 5,000 species (Shear et al., 2016). Family representatives include bioluminescent species, brightly colored aposematic species, and taxa that make up widespread Müllerian mimicry rings in the Appalachian Mountains (Marek & Bond, 2009; Marek & Moore, 2015). Despite the fascinating biological aspects of this millipede order and number of undescribed species, the taxon has been difficult to place at the ordinal level, and the relationships within Polydesmida are *terra incognita* (Sierwald et al., 2003; Shear, 2008; Blanke & Wesener, 2014; Fernández, Edgecombe & Giribet, 2016). Lack of a systematic framework for Polydesmida at all taxonomic levels has hampered investigation of this very interesting yet understudied taxon, and the basic alpha-taxonomic descriptions of species lags well behind other better-known invertebrates by 50–100 years. The diversity and geographical distribution of the Xystodesmidae and its similarity with Plethodontidae suggests an early divergence possibly during the K-T or early Tertiary (Shen et al., 2016). Like plethodontid salamanders, xystodesmids have a center of diversity in Appalachia; however, Shen et al. (2016) indicated that plethodontid salamanders have an origin of diversity in the western US with high species diversification in Appalachia associated with the genera *Plethodon* Tschudi, 1838 and *Desmognathus* Baird, 1850 during the Miocene. These dates may be consistent with the family Xystodesmidae but remain untested in the taxon.

**Figure 1:**
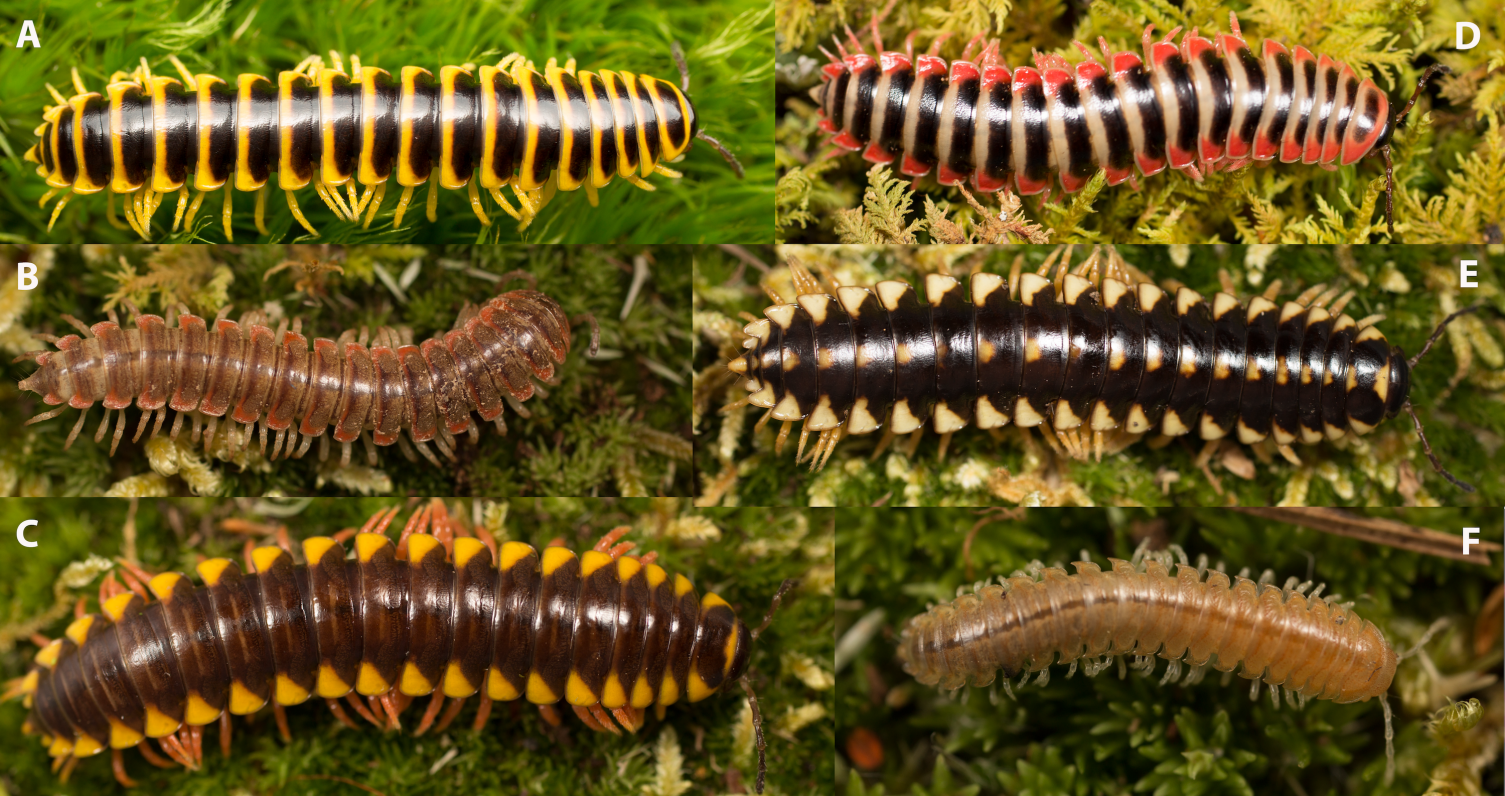
Photographs of various xystodesmid species showing a variety of color patterns. Dorsal view of six adult male Xystodesmidae. (A) *Sigmoria whiteheadi*; (B) *Dicellarius atlanta*; (C) *Appalachioria* n. sp. ‘Clinch Mountain’; (D) *Sigmoria nantahalae*; (E) *Appalachioria* n. sp. ‘Clinch Mountain’; (F) *Gyalostethus monticolens*. (C) and (E) were found in the same locality near Mendota, VA, which had at least 12 color morphs in the same population.

Individuals of the family Xystodesmidae are diagnosed by a large body size—ca. 40 mm—glossy and colorful dorsal surface, reduced segmental appearance, simple, usually undivided gonopods, and the presence of prefemoral spines (Fig. 1) (Marek, Tanabe & Sierwald, 2014). While these are the general diagnostic features of the family, morphological limits and the monophyly of the taxon remain untested, and these characters have never been evaluated within a phylogenetic context. Over the 120-year history of the family, its taxonomy has been built almost entirely on male genitalic variation (Cook, 1895; Brölemann, 1916; Attems, 1926; Hoffman, 1962; Shelley & Whitehead, 1986). Male polydesmidan millipedes, including Xystodesmidae, have paired genital openings of the vas deferens on the third body ring onto which a pair of legs on the seventh body ring modified for intromittent copulation called gonopods are placed and filled with sperm directly before insemination of the female (Tanabe & Sota, 2008). Taxonomic treatments of nearly all Diplopoda have focused on male genitalic characters, due to a generally conservative somatic morphology (perhaps due to streamlining for their burrowing habits) and the presumed importance of gonopod genitalic shape in the enforcement of reproductive isolation.

While likely important for the enforcement of reproductive isolation, particularly in blind xystodesmid millipedes where species recognition cues are limited, the use of gonopod variation as the basis for phylogenetic analyses is problematic for multiple reasons. Paramount among them is the paucity of information about the drivers of gonopodal variation. While multiple factors could be at play, including reproductive character displacement (Bond & Sierwald, 2002; Kameda, Kawakita & Kato, 2009), there are two popular theories as the primary driver of genitalic variation in millipedes: (1) lock-and-key (Dufour, 1844) and (2) rapid and divergent variation as a result of female choice (Eberhard, 2010). Under lock-and-key theory, female genitalic form (the lock) matches male genitalic form (the key) and serves for species recognition via mechanical means. Such an evolutionary mechanism involves stabilizing selection and genitalic co-evolution between the sexes as both the “lock” and the “key” would need to change in tandem to maintain mechanical fit between them. The second mechanism for genitalic variation in millipedes involves sexual selection via female choice (SS). In SS, mating success is disproportionately controlled by one sex, who may refuse to mate, break off mating mid-way or (in the case of the female) perhaps eject, destroy or dilute the male sperm post-copulation. Because there is no overt manifestation of the stimulatory threshold of acceptance by the female, choice is generally recognized as cryptic and the mechanism is referred to as sexual selection via cryptic female choice (Eberhard, 1996). The cryptic nature of SS hinders investigation, making comparisons of SS and lock-and-key challenging. However, a small number of authors have undertaken this task and found evidence for lock-and-key or neutral/pleiotropic evolution of gonopod shape (Wojcieszek & Simmons, 2012; Bond et al., 2003; Tanabe & Sota, 2008). The research is ongoing, and further studies are needed before a general understanding of the drivers of genitalic variation can develop, especially if multiple mechanisms could be at play.

Although the causes of genitalic variation in millipedes are uncertain, gonopodal variation tracks geographically and genetically cohesive lineages, as evidenced by the high degree of male gonopodal variation between related species of xystodesmid millipedes. For example, in a clade of *Brachoria* species from the Cumberland Mountain Thrust Block, closely related species exhibit prominent shape differences between gonopods (Marek, 2010, cf. fig. 38). The genitalic differences provide a straightforward basis for naming new species (but see the tautology discussed by Bond et al., 2003). Though useful for species-level alpha-taxonomy, pronounced morphological differences between closely related species mask synapomorphies among higher-level taxa. In a treatment of the xystodesmid genus *Sigmoria* by Shelley & Whitehead (1986), the authors estimated a phylogeny of the eastern tribe Apheloriini that contains many of the Appalachian species and referring to gonopod characters noted, “[n]early all of the characters and character conditions that we presently have for the analysis are homoplasious” and “major gonopodal differences may be evidence for nothing more than species level differences.”

While genitalic characters are a premier source of highly divergent features to differentiate species, they appear problematic above the species level for use in the classification of the family Xystodesmidae (Shelley & Whitehead, 1986). Incorporating multiple data sets including DNA sequence characters with morphology, in an integrative analysis, has been demonstrated to be informative to assess agreement between datasets, a common phylogenetic signal, and to reach a consensus on classification (e.g., Pimvichai, Enghoff & Panha, 2014; Enghoff, Petersen & Seberg, 2011; Will, Mishler & Wheeler, 2005). Another dataset for systematics in low-mobility dispersal-limited arthropods, including xystodesmid millipedes, is geographic proximity (Hedin et al., 2012; Marek, 2010; Bond & Stockman, 2008; Shelley & Whitehead, 1986). When finding gonopod characters to be generally homoplasious, Shelley & Whitehead (1986) integrated (1) geographical proximity and (2) morphology, primarily gonopod structure, to refine their hypothesis from their initial one (Shelley & Whitehead, 1986). Without cladistics and using a primarily heuristic method, Shelley & Whitehead (1986) accomplished this by first weighting select morphological characters to unite taxa to produce a phylogeny (preliminary hypothesis, cf. pg. 196), and subsequently adjusted this phylogeny with geographical data (by uniting taxa that shared geographical regions) to produce a revised phylogeny (cf. pg. 200). Shelley & Whitehead (1986) argued that inclusion of geographic distribution data improved phylogenetic accuracy in the family Xystodesmidae. Xystodesmid millipedes are generally restricted in distribution and phylogeographic analyses show restricted gene flow with isolation by distance along linear mountain ranges in Appalachia (Marek & Bond, 2009). This indicates that geographical and genetic variation are significantly associated with one another. Therefore, geographical proximity should be a good predictor of evolutionary relationships in the family. While there is a general lack of studies explicitly testing whether geographic distributions of closely related low-mobility taxa can better predict phylogeny than can morphology, one such investigation which tests this hypothesis is that of Hedin et al. (2012). The authors found that geography closely predicts monophyletic clades at the regional and continental-scale geographic level within the sclerosomatid Opiliones.

When beginning this study, we sought to determine the evolutionary history of a state-threatened microendemic species, the Laurel Creek Millipede *Sigmoria whiteheadi*. Notably, *S. whiteheadi* is extremely limited in distribution with its global range restricted to less than a one-kilometer squared area. Somatically, *S. whiteheadi* is similar to many millipedes of the genus *Sigmoria*, with bright yellow stripes and a glossy black cuticle, and the species was placed in the *rubromarginata*-group of the genus based on curvature and the ribbon-like distal end of its gonopod (Shelley & Whitehead, 1986, pg. 105). For 30 years, the species had not been recollected despite the efforts of R.L. Hoffman to find additional populations. To provide an accurate and detailed assessment of this rare species, we conducted a thorough survey of *S. whiteheadi* to document its distribution. We conducted the survey because it is a state threatened species in Virginia (Roble, 2016), extremely restricted geographically, and possesses conservation value for preserving the natural heritage of Virginia. As this is the case with many species of Xystodesmidae, and many are known from a single type locality (Marek, 2010), we sought to provide an in-depth and systematic survey of *S. whiteheadi* in order to address this general phenomenon of very restricted distributions in the family. Based on results presented here, and consistent with the theme of gonopodal homoplasy, *S. whiteheadi* is unexpectedly more closely related to species in the genus *Apheloria*. This was unforeseen given the shape of its gonopods, which are robust and “sigmoid-shaped,” an apomorphy historically proposed to unite species of *Sigmoria*, and not thin and circular as is uniformly the case in *Apheloria* (Shelley & Whitehead, 1986).

Based on the relevance of *S. whiteheadi* in conservation, and observations of rampant gonopodal variation across Xystodesmidae (seemingly inconsistent with phylogeny), we tested the hypothesis that genitalic characters are not better than geographical distance in recovering an accurate phylogeny. To test this question, we inferred the largest molecular phylogeny of Diplopoda to date with 90 species-group taxa within the family Xystodesmidae and nucleotide data from six gene regions (∼4,000 bp). We inferred trees based on: (1) a matrix assembled from published morphological characters and (2) geographic proximity (Euclidean distance) of species. These two trees were then the basis of several topology-based comparisons against the molecular phylogeny. This paper accomplishes three objectives: (1) an estimation of the molecular phylogeny of the Xystodesmidae, (2) a test of whether geography is an accurate predictor of phylogeny within the family, and (3) a determination of the phylogenetic placement and geographic distribution of the Laurel Creek Millipede, *S. whiteheadi*.

## Materials and Methods

### Taxon sampling

Molecular partition. Exemplar specimens of all species used in this study were collected from 2003 to 2015, of which ca. 51% were from original type localities (Appendix A). We targeted species in the tribe Apheloriini, which historically contained *S. whiteheadi*, and two other closely related tribes of xystodesmid millipedes based on the phylogeny from Marek & Bond (2006) and Marek & Bond (2007): the Pachydesmini and Rhysodesmini. Of the 17 nominal genera in Apheloriini, we included 13 representatives in the phylogeny. In addition, we included genera from the Pachydesmini (*Boraria*, *Cherokia*, *Gyalostethus*, *Pleuroloma* and *Stenodesmus*) and Rhysodesmini (*Dicellarius* and *Pachydesmus*) as a diverse outgroup selection. Species were represented by male specimens since the male gonopods are almost exclusively used as identification resources to the species level. These species represent 61% of the 116 described species in Apheloriini, along with six undescribed species.

Morphological partition. Specimens that were used for the molecular partition were scored for morphological characters (Appendix B). We used the morphological characters from Marek & Bond (2006), Marek & Bond (2007), and Shelley & Whitehead (1986), to develop a character matrix and score an additional 29 species for a total of 68 morphological characters. Both male and female specimens were examined in the morphological partition. Female specimens, which are challenging to identify below the genus level because they lack species-characteristic gonopods, were identified based on criteria delineated by Marek (2010). Characters from both sexes of a single species were combined into one terminal in the morphological character partition.

### Specimen collection and curation

Specimen collection and processing techniques are those described in Means et al. (2015). Millipedes collected in the field were brought to the lab alive for habitus photography, preservation of genetic material, and storage in the Virginia Tech Insect Collection (VTEC, http://collection.ento.vt.edu/). Legs posterior to the gonopods (#10–22) were removed from the left side of each specimen and stored in RNA*later* at −80 °C for archival preservation of RNA and DNA (Qiagen, Hilden, Germany). DNA was extracted using a Qiagen DNeasy tissue kit. In the rare case where legs were removed from specimens stored in 100% EtOH, the appendages were air-dried prior to DNA extraction. Typically three legs were used for large millipedes (most Xystodesmidae), while 4–6 legs were used for smaller millipedes (e.g., *Stenodesmus tuobitus* and *Gyalostethus monticolens*). Extracted and purified DNA was stored at −20 °C.

Six gene fragments in the following regions were amplified for each specimen: large subunit ribosomal RNA gene including the tRNA-Val gene (16S), small subunit ribosomal RNA gene (12S), 28S ribosomal RNA gene (28S), cytochrome c oxidase subunit I gene (COI), and elongation factor-1 alpha gene (EF1-a). Amplification procedures can be found in Appendix C. Amplified DNA was cleaned, concentration quantified and normalized, and sequenced at the University of Arizona Genetics Core using an Applied Biosystems 3730 DNA Analyzer (Applied Biosystems, Foster City, CA, USA).

### Sequence alignment and phylogenetic inference

Sequence chromatograms were edited in the Mesquite module Chromaseq (Version 1.2) implementing phred and phrap for nucleotide base-calling, trimming, and quality control (Maddison & Maddison, 2010; Ewing et al., 1998). Sequences for the six genes were exported as individual FASTA files to PRANK (Version 140110) for multiple sequence alignment (Löytynoja & Goldman, 2005). In PRANK, the default iterative process of constructing multiple guide trees was used, and -F option was included for the mitochondrial ribosomal genes where there was a large number of insertions/deletions. Aligned sequences were partitioned by gene, codon position, and exon/intron boundaries, and concatenated in Mesquite. PartitionFinder (Version 1.1.1) was used to compare alternative partitioning schemes and nucleotide substitution models using the Bayesian Information Criterion (BIC) model selection method and models of evolution for MrBayes (Lanfear et al., 2012). MrBayes (Version 3.2.2) was used for phylogenetic inference with the best-fit partitioning model (Ronquist et al., 2012). We ran two hot and two cold independent MCMC chains simultaneously, retaining two of the chains for an initial 1,000,000 generations each. Each chain was sampled every 100 generations, with a one-quarter generation burn-in to reach a posterior distribution of trees and a consensus topology. The standard deviation of split frequencies (SDSF) convergence statistic was monitored during the analysis and then checked upon completion to ensure convergence between the independent chains (Ronquist, Huelsenbeck & Van der Mark, 2005). Upon conclusion of the run, parameters were averaged and the consensus topology generated from the posterior distribution of trees with a consensus type of all compatible groupings. To assess varying resolutions of gene histories, single-gene trees were independently estimated in MrBayes. Nucleotide frequencies and base composition were assessed in PAUP* (Swofford, 2002).

### Morphological character coding, scoring and phylogenetic analysis

Ninety xystodesmid millipede species-group taxa were examined for 68 qualitative morphological characters (Appendix B). Morphological traits were examined and character states scored using a Leica M125 stereomicroscope (Leica Microsystems, Wetzlar, Germany). Gonopods from the left side of the 7th body ring were dissected following Means et al. (2015), and photographed in alcohol with a Canon 6D dSLR camera and a 65 mm MP-E macro lens mounted on a Passport II Portable Digital Imaging System (Visionary Digital, Charlottesville, VA, USA). Gonopods were photographed at 6–20 focal planes and stacked in the program Helicon Focus (Helicon, Kharkiv, Ukraine). Gonopods were illustrated by tracing photographs of specimens in Adobe Illustrator CS6 (Adobe, San Jose, CA, USA). The matrix was comprised of 46 binary and 22 multi-state characters, of which 40 were male gonopodal characteristics, 23 were from the exoskeleton and five were from female cyphopods (Shear, 1972; Shelley, 1981; Shelley & Whitehead, 1986). The morphological dataset was assembled and characters scored in Mesquite. MrBayes was used to infer a phylogeny based on the morphological data partition using likelihood-based models of character state change. Tree topologies were evaluated based on the Markov k (Mk) model (Lewis & Puterman, 2001) with and without Γ-distributed rates of character change, and with the character coding set to “variable”. We ran the analysis as with the molecular partition, monitoring the SDSF diagnostic to ensure convergence and averaging the parameters and generating a consensus tree with all compatible groupings. Alternative models with and without Γ distributed rate variation were compared with Bayes factors to determine the best-fit to the data.

### Homoplasy analysis

To assess homoplasy of morphological characters and therefore evaluate utility of the features as diagnostic characters for taxonomy, character states were mapped onto the molecular phylogeny using the posterior probability mapping program SIMMAP 1.5.2b21072010 (Bollback, 2006). SIMMAP takes into account the uncertainty in modeling evolutionary change, using parameters such as branch length, rates of evolutionary change and topology (Bollback, 2006; Huelsenbeck, Nielsen & Bollback, 2003). To map morphological characters onto the molecular tree, we used a morphological/standard model with a bias parameter and a beta distribution prior (alpha = 1, *k* = 31) for binary characters and an empirical prior for multistate characters (Bollback, 2006). We ran a Markov chain Monte Carlo analysis with 100,000 generations with a 10% burn in for each morphological character to determine the best-fit rate parameter alpha and beta priors (*k* = 90, *T* rate = 1). To measure homoplasy, the number of transformations per character were estimated using SIMMAP and then used to calculate the consistency index (CI) for each character, calculated: CI = expected number of state changes/observed number of state changes. The degree of homoplasy for a morphological character (HI = 1–CI) decreases as the CI approaches one. SIMMAP was also used to calculate the dwell time for each character state, which is the proportion of time that a character spent in a specific state (Bollback, 2006).

Because male genitalia are highly variable anatomically and central to the taxonomy of the family, we evaluated the spatial distribution of homoplasy on the gonopods. Male gonopods are composed of six podomeres that have fused during gonopod development, and only three vestigial podomeres, the coxa, prefemur, and tibiotarsus, (the latter of which is a fusion of the other four segments) remain distinguishable. We divided the gonopod into these three regions and summed the CI in each region (dividing each region’s sum by the total of all three regions to standardize) to assess if CI, and convergence, is evenly spread among the podomeres.

### Geographical distance tree

Geographic distributions for each taxon were derived from published taxonomic records and material from the VTEC, databased in the biodiversity data manager Symbiota Collections of Arthropods Network (SCAN, symbiota4.acis.ufl.edu/scan). Many of the taxonomic collection localities in the literature were narrative in nature and did not include precise geographical coordinates. For these, collection localities were manually georeferenced in Google Earth (Google, Mountain View, CA, USA). These data are available for download from VTechData (https://data.lib.vt.edu/files/qz20ss52b). Geographical coordinates were plotted in ArcGIS (ESRI, Redlands, CA, USA), converted into individual shapefiles according to species, and species centroids calculated. Species whose distribution centroids fell within the same Level III Ecoregion (Omernik, 1987) were combined into a group designated by that ecoregion. Since xystodesmid species distributions are often <20 km^2^ and densely packed into many parapatric distributions in a single area, i.e., the “mosaic distributions” of Shelley & Whitehead (1986), using species centroids to determine membership within an ecoregion was robust to errors associated with potentially arbitrary placement of centroids of widely-distributed species. Distance between Level III Ecoregion centroids were calculated in the Geographic Distance Matrix Generator (Ersts, 2011, version 1.2.3), which uses the curvature of the Earth to determine the geographic distance between points according to surface convexity, and outputs a distance matrix for further use (Ersts, 2011). This matrix was input into PAUP* (Swofford, 2002, version 4.0a150) to calculate a neighbor-joining tree based on the centroid distance matrix between species.

### Bayes factor comparisons of alternative trees

To compare alternative hypotheses of topology, the molecular tree was constrained to each of the alternative tree-hypotheses in the program MrBayes using the “constraint” command and an absolute constraint prior of one. The alternative topologies that were assessed were (1) the ecoregion distance tree, (2) the prior phylogeny of Shelley & Whitehead (1986), and (3) the morphology tree inferred based on the matrix assembled in this study. The topology of Shelley & Whitehead (1986) (hereafter referred to as “SW86”) did not assess species-level relationships and therefore was resolved at the genus-level with 11 constraints (Shelley & Whitehead, 1986, in text Figs. 156, 160). The ecoregion topology (hereafter referred to as “EDT”) had eight constraints, one for each of the ecoregions in which species centroids occurred: Piedmont, Southeastern Plains, Blue Ridge, Ridge and Valley, Southwestern Appalachians, Central Appalachians, Western Allegheny Plateau and Interior Plateau (Omernik, 1987). Both the morphological and molecular topologies (hereafter referred to as “MRT” and “MOT”) had 89 constraints, representing the fully resolved branching pattern of each tree. We used the stepping-stone search algorithm in MrBayes to estimate the marginal likelihoods of each of the constrained trees for Bayes factor comparisons (Bergsten, Nilsson & Ronquist, 2013; Xie et al., 2011). The stepping-stone (SS) analysis separates the tree search into a series of independent MCMC chains. As the “steps” progress from the posterior distribution to the prior distribution, the ratio of trees sampled from each varies. Additionally, each step serves as the burn-in for the subsequent step. SS has been shown to be more accurate than harmonic mean (i.e., MCMC) and thermodynamic integration methods (Xie et al., 2011) when calculating likelihoods for Bayes factor comparisons. Each SS analysis included 50 steps and resulted in a mean marginal likelihood value, with the highest value indicating the marginal likelihood of the posterior distribution of trees. The molecular data were constrained to the MOT to produce a likelihood value, which could be compared to the other constrained topologies. We used the difference between mean marginal likelihood value for the constrained analysis versus the MOT to assess the varying explanatory powers of the alternative hypotheses, and whether geographical proximity was a better predictor of phylogeny than the prior phylogeny of SW86.

### Survey and phylogenetics of the Laurel Creek Millipede, *Sigmoriawhiteheadi*

In order to determine the distribution of *S. whiteheadi*, collections were carried out every 0.5 km in linear transects radiating from the four cardinal directions from the type locality, for a total of 32 sample locations (National Park Service Permit # BLRI-2014-SCI-0033). Sampling protocols followed Means et al. (2015). A maximum of five adult individuals were retained from each sampling locality to lessen disturbance to the population. Additional fine-scale collecting was performed on the periphery of the known distribution until individuals were no longer encountered. Live specimens were brought back to the lab, photographed and processed as described above and in Means et al. (2015). DNA was extracted and six gene fragments (COI, EF1a, 12S, 16S, 28S and tRNA-Val) were then sequenced to infer evolutionary relationship within the phylogeny of Xystodesmidae.

## Results

### Sequence alignment and phylogenetic inference

Sequences of the gene regions were separately aligned in PRANK using the HKY substitution model with empirical base frequencies and a kappa =2, which resulted in a total aligned length of 3,975 bp for the concatenated supermatrix as follows: COI (1–600), 12S (601–749), tRNA-Valine (750–830), 16S (831–2,266), 28S (2,267–3,351) and EF1a (3,352–3,975). The alignment was made up of six gene regions subdivided in PartitionFinder into seven partitions (Table 1). Of the 3,975 characters, 2,566 were constant, 478 were parsimony uninformative and 931 were parsimony informative. Observed mean base pair composition for the concatenated matrix was *A* = 0.21579, *C* = 0.171, *G* = 0.26965, *T* = 0.34355. Nucleotide frequency was homogenous across taxa for each gene region (*P* > 0.05, *x*^2^ > 12.47).

**Table 1:** Partitions used in MrBayes phylogenetic analysis of molecular data.

Partition	Gene region	Best-fit models	PIC
1	CO1 (1st CP)	GTR + I + Γ	28
2	CO1 (2nd CP), EF1a (1st & 2nd CP)	HKY + I + Γ	11
3	CO1 (3rd CP)	GTR + I + Γ	168
4	12S, tRNA-Valine, 16S	GTR + I + Γ	564
5	28S	GTR + I + Γ	62
6	EF1a (3rd CP)	HKY + I	45
7	EF1a (intron)	HKY + Γ	53

**Notes.**

PIC Parsimony Informative Characters CP Codon Position

**Figure 2:**
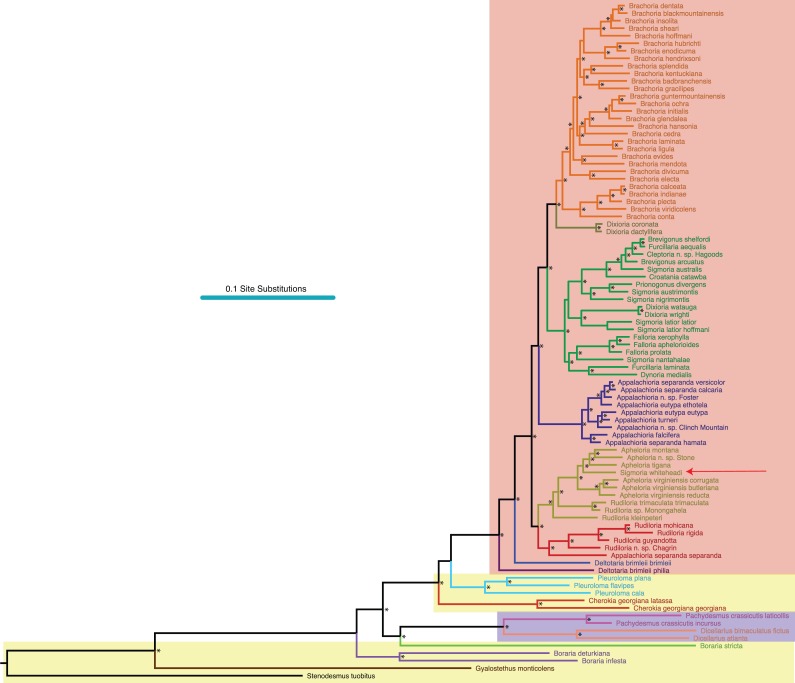
Molecular phylogeny of Xystodesmidae. Asterisks indicate a posterior probability of >0.70. Colored boxes indicate tribes: yellow, Rhysodesmini; purple, Pachydesmini; red, Apheloriini. Color branches and names indicate monophyletic groups that potentially warrant unique generic status: black, *Stenodesmus*; brown, *Gyalostethus*; light purple, *Boraria*; light green,* Boraria stricta*; peach, *Dicellarius*; pink, *Pachydesmus*; burnt sienna, *Cherokia*; turquoise, *Pleuroloma*; dark purple, *Deltotaria brimleii philia*; blue, *Deltotaria brimleii brimleii*; red, *Rudiloria*; gold, *Apheloria*; navy, *Appalachioria*; green, sigmoid clade; mustard, *Dixioria*; orange, *Brachoria*. The placement of *Sigmoria whiteheadi* within the *Apheloria* is indicated by a red arrow.

MCMC chains were run for 1 M generations and likelihood values converged after 315,000 generations. Of the trees generated, after one-quarter were discarded as burnin, 15,000 in the posterior distribution were summed for the consensus tree (Fig. 2). The molecular phylogeny from the concatenated dataset of five gene regions recovered a well-supported topology with high posterior probability node support values where 71 of 88 nodes were ≥0.95 (Fig. 2). The tribes Apheloriini and Pachydesmini are recovered as monophyletic; however, the tribe Rhysodesmini is not monophyletic and occurs as a paraphyletic grade at the base of the Apheloriini. The genera *Brachoria*, *Cherokia*, *Dicellarius*, *Falloria*, *Pachydesmus*, and *Pleuroloma* were recovered as monophyletic (Fig. 2). In contrast, the genera *Apheloria*, *Appalachioria*, *Boraria*, *Brevigonus*, *Deltotaria*, *Dixioria*, *Furcillaria*, *Rudiloria*, and *Sigmoria* were found to be polyphyletic. *Apheloria* is recovered as paraphyletic with respect to *S. whiteheadi* (posterior probability node support [*pp*] = 0.84), and that clade in turn renders *Rudiloria* paraphyletic (*pp* = 0.98). *Appalachioria* is a monophyletic clade with the exception of *A. s. separanda* that occurs sister to *Rudiloria* + *Apheloria*. *Boraria infesta* and *B. deturkiana* are sister species (*B. profuga* was not included in this study); in contrast *Boraria stricta* is recovered as a sister group to the Pachydesmini (*pp* = 0.89). *Brevigonus* is paraphyletic due to the placement of *Furcillaria aequalis* as sister to *Brevigonus shelfordi* (*pp* = 1). The two subspecies of *Deltotaria brimleii* are a paraphyletic grade at the base of the Apheloriini (*pp* = 1). *Dixioria* is recovered as polyphyletic with *Dixioria wrighti* and *D. watauga* forming a monophyletic clade within the greater sigmoid-gonopod clade (*pp* = 0.75) and *D. coronata* and *D. dactylifera* representing a monophyletic clade sister to *Brachoria* (*pp* = 1). *Furcillaria* is polyphyletic with *F. aequalis* sister to *Brevigonus shelfordi* and *Furcillaria laminata* sister to *Dynoria medialis* (*pp* = 1). *Rudiloria* is polyphyletic and includes a clade of northern species: *Rudiloria guyandotta*, *Rudiloria mohicana*, *Rudiloria rigida*, including an undescribed species from Ohio. The species *Rudiloria kleinpeteri*, *Rudiloria trimaculata trimaculata* and a newly discovered unnamed species of *Rudiloria* from Monongahela National Forest are separate from the northern species and paraphyletic with respect to the genus *Apheloria*. *Sigmoria* is recovered as polyphyletic, with *Sigmoria nantahalae* as sister to the monophyletic *Falloria* (*pp* = 0.97). The representatives of the *Sigmoria latior* complex are monophyletic and are sister to *Dixioria wrighti* and *D. watauga* (*pp* = 1). *Sigmoria austrimontis* is sister to *Prionogonus divergens* (*pp* = 1), and *Sigmoria australis* is sister to the *Brevigonus* species (*pp* = 1).

The independent gene trees showed a high degree of concordance at deep phylogenetic divergences and some discordance at a shallow level. The ribosomal 16S gene region most closely resembled the consensus tree and included a monophyletic *Pachydesmus*, *Pleuroloma*, *Cherokia*, and *Brachoria*; a paraphyletic *Boraria*, *Appalachioria*, *Dixioria,* and *Brevigonus*; and a polyphyletic *Sigmoria*, *Rudiloria* and *Apheloria* (Appendix D). The trees for COI, 28S and EF1a were more variable, recovering only *Pleuroloma* as monophyletic throughout, though *Falloria* was recovered as monophyletic in 16S, 28S and EF1a. *Sigmoria whiteheadi* was recovered as a member of *Apheloria* in 16S and COI, but as sister to *A. eutypa eutypa* in EF1a. The species was not included in the 28S matrix due to the low phred scores of the 28S chromatograms, and therefore unalignable sequence.

### Morphological character coding, scoring and phylogenetic analysis

**Figure 3:**
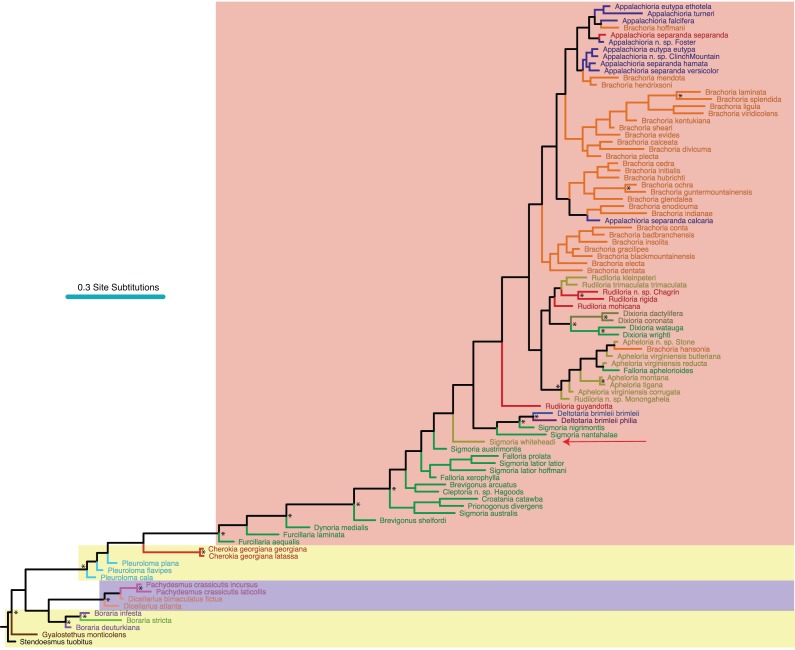
Morphological phylogeny of Xystodesmidae. Asterisks indicate a posterior probability of >0.70. Colored boxes indicate tribes, and colored branches/names indicate genera, as in Fig. 2. The placement of *Sigmoria whiteheadi* is indicated by a red arrow.

Of the 68 characters used in the morphological analysis, 67 were parsimony-informative. We implemented the Mk + Γ model of character change due to “very strong (BF > 10)” evidence against the simpler Mk model (Bayes factor of 118.95, Kass & Raftery, 1995). Convergence of the Mk + Γ analysis occurred after 22.6 M generations. Of trees generated, one-quarter were discarded as burnin and 48,000 remaining in the posterior distribution were summed for the consensus tree (Fig. 3). The morphological phylogeny from the 68 variable characters recovered a monophyletic *Boraria,* sister to a monophyletic Pachydesmini, as well as a monophyletic *Cherokia*, *Deltotaria* and *Dixioria* (Fig. 3). *Dicellarius* is rendered paraphyletic and at the base of the Pachydesmini, as are *Furcillaria* and *Brevigonus* at the base of the Apheloriini (Fig. 3). *Brachoria* includes *Appalachioria* but is rendered paraphyletic by *B. hansonia*, which is outside of *Brachoria* and with species of *Apheloria*. *Rudiloria* species (spare *R. guyandotta* and the unnamed species of *Rudiloria* from Monongahela N.F.) are monophyletic and sister to *Dixioria*. *Pleuroloma, Falloria, Sigmoria* and *Apheloria* are all polyphyletic in the MRT. *Sigmoria whiteheadi* is recovered as a sister species to a large clade of apheloriine species, and not close to *Apheloria.*

### Bayes factor comparisons of alternative trees

The previous hypothesis of phylogeny (SW86) was the closest to the molecular tree (MOT), with a Bayes Factor (BF) score of 874.11. The EDT tree based on ecoregions had a BF score of 4,841.18, versus the MRT (morphology tree) that was the most divergent from the MOT with a BF score of 4,941.06. For all four stepping-stone analyses, 50 steps were performed and the first step was discarded as burn-in, with each step acting as the burn-in for the subsequent step. The molecular and morphological constraint analyses both converged during the second step in the stepping stone analysis (25,000 generations) and had 19,600 generations per step. The SW86 analysis converged after 43 steps and had 1,254,900 generations per step, while the EDT analysis converged after 44 steps and had 1,254,900 generations per step.

### Homoplasy analysis

**Figure 4:**
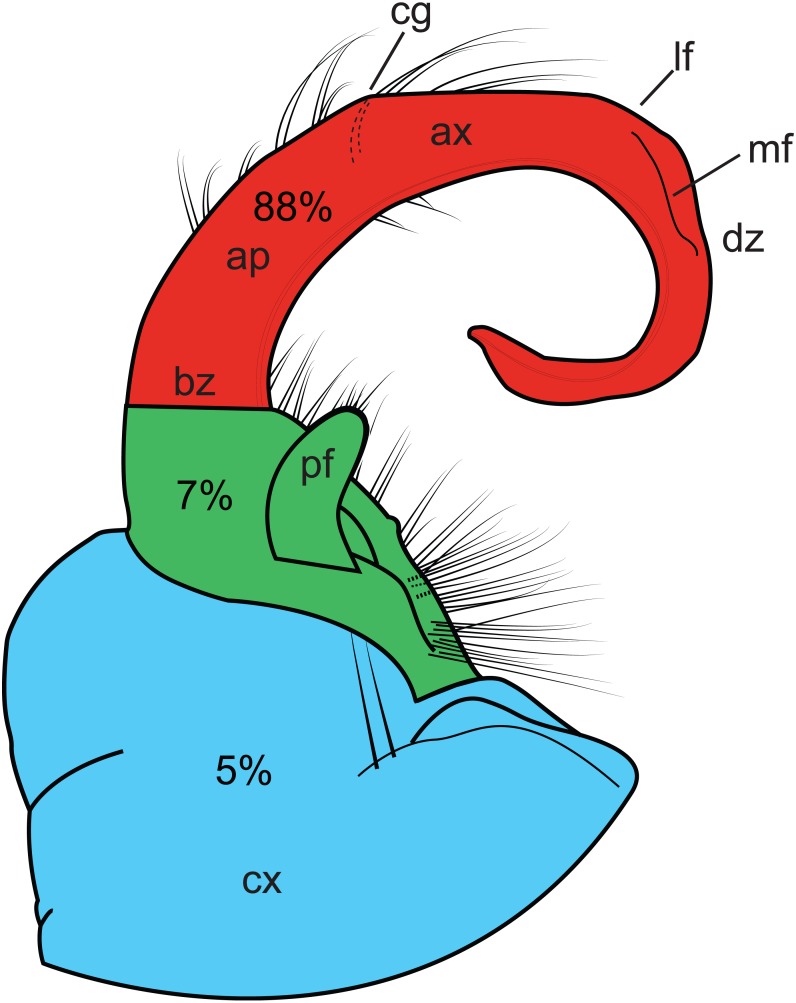
Illustration of the left gonopod of a male *Sigmoria whiteheadi*. Colors and labels indicate different gonopodal regions. Light blue, coxa; light red, tibiotarsus; green, prefemoral region. Ap, acropodite; ax, apex; bz, basal zone; cg, cingulum (not present on *S. whiteheadi*); cx, coxa; dz, distal zone; lf, lateral flange (found on opposite side of medial flange); md, medial flange; pf, prefemoral process. Percentages relate to the amount of overall homoplasy found on all 90 gonopods scored.

Homoplasy index values ranged from 0.338 to 0.991. Figure 4 graphically depicts the relative amount of homoplasy among three regions of a generalized gonopod: the coxa, the prefemur and the tibiotarsus (HI values are colored as a heat-map). The tibiotarsus (Fig. 4, section 3, in red) showed the highest average homoplasy, accounting for 88% of all gonopodal homoplasy, with 5% and 7% associated with coxal and prefemoral characters, respectively. Individual homoplasy indices and dwell times for each character can be found in Appendix B.

### Short-range endemism and the state threatened Laurel Creek Millipede, *Sigmoria whiteheadi*

After 30 years, the Laurel Creek Millipede was rediscovered near its historical type locality in Virginia (Fig. 1A). The historical distribution of *S. whiteheadi* as a single locality where the type series was collected in 1983 is now expanded and includes eight additional sites—for a total of nine—all of which occur within a slender, linear area of 0.95 km^2^. All individuals of *S. whiteheadi* were found within a thin 2-km long linear rhododendron and hardwood dominated riparian border of Laurel Creek, extending 1 km north and 1 km south from the type locality. Adult individuals were predominantly found under fallen oak (*Quercus* spp.), maple (*Acer* spp.) and tulip poplar (*Liriodendron tulipifera*) leaves in small broadleaf forests bordered by rhododendron. In contrast, immatures were found predominantly beneath rhododendron leaves within the rhododendron corridor of the stream.

## Discussion

### Comparison of geographical and morphological topologies

There was very strong evidence that the alternative topologies (SW86, EDT and MRT) were all highly distinct from the molecular tree, MOT (BF ≥10; Kass & Raftery, 1995), indicating that neither morphology nor geography are good predictors of evolutionary relationships when used individually. However, SW86 was most consistent with the molecular phylogenetic hypothesis (MOT versus SW86, BF = 874.11). While the SW86 phylogeny is most consistent with the MOT, there are some major differences. These differences include the concept of *Sigmoria* and monophyly of several apheloriine genera. While the MOT is consistent with a widespread morphologically complex (in terms of gonopods) *Sigmoria*, as Shelley & Whitehead (1986) hypothesized, their subgenera are not monophyletic, except for *Falloria*—*Sigmoria nantahalae* had since been removed from the genus *Falloria* and placed in *Sigmoria* (Hoffman, 1999). (Notably, all the species of *Falloria* have an eggplant-purple or white metatergal stripe, which is markedly distinct from the other genera in the sigmoid clade.)

The conformity of the SW86 and MOT may support the methodology of prior traditional phylogenetic hypotheses that integrated geographical proximity and morphology over reliance on geographical or morphological data alone to determine the placement of most higher-level taxa. Direct comparisons between the MRT, SW86, EDT, and MOT phylogenies based on their Bayes factor scores alone is challenging, primarily due to the disparity between the number of constraints for each topology. However, the EDT and SW86 topologies possessed about the same number of constraints, and are most easily compared. With fewer constraints in the SW86 and EDT topologies, the phylogenetic analyses were less constrained to vary and relied on the phylogenetic signal in the molecular data for taxon placement. However similar in number of constraints, the significant disparity between the Bayes factor for the SW86 and EDT topologies (874.11 vs. 4,841.18, respectively) is unlikely due to the small difference in number of constraints and instead indicative of the varying power of the phylogenetic signal in each dataset.

The approach to use geographical proximity alone to assemble clades did not significantly improve upon the prior phylogeny of Shelley & Whitehead (1986), indicating that using solely geography is not a good predictor of evolutionary history of xystodesmid genera. Basic geographical distance alone as a source of evolutionary relatedness is problematic, especially in older groups, as xystodesmid millipedes are likely to have diversified in the Appalachians during the Miocene (that is if congruent with co-distributed taxa such as plethodontid salamanders) and the distributions of higher level taxa, such as genera, will have had ample time to expand and contract, thereby making reconnections between distantly related groups more probable. The movements of taxa over long periods of time likely established areas of sympatry between distantly related taxa thereby confounding the use of geographical proximity as a sole indicator of evolutionary relatedness. However, integrating geographical proximity for the prediction of evolutionary relatedness below the genus level, and between closely related taxa, may remain useful. Perhaps especially in groups with low dispersal capabilities like xystodesmid millipedes. For example, studies implementing Templeton-Crandall-Sing parsimony and inferring demographic processes using nested clade analysis in millipedes, have generated hypotheses useful for conservation and understanding phylogeographic patterns (Zigler, Loria & Lewis, 2011; Marek & Bond, 2009). However, in very old groups of non-mobile invertebrates, the technique of using geographical proximity is more challenging (Hedin & McCormack, 2017; Hendrixson & Bond, 2007).

While geographical proximity may be useful to inform relationships between recently diverged species, morphological similarity (at least in millipede gonopods) is not. Convergence of male genitalic features can complicate the understanding of species-level systematics in the Xystodesmidae. For example, the cingulum of *Brachoria* and *Appalachioria* is now known to occur in three different parts of the tree. As shown here, 95% of the gonopodal characters used in traditional xystodesmid systematics are homoplasious (HI > 0.5). Not surprisingly, the most homoplasious characters were relatively subjective characters, such as the overall shape of the gonopophyses (male openings of the vas deferens) and presence of lateral flanges, which are variously located on different regions of the gonopod (Fig. 4). One of the least homoplasious characters is the presence or absence of a “gonopodal fold”, which is essentially a cingulum, and is the diagnostic character for genus *Cherokia*. However, the gonopodal fold was never inferred to be homologous with the cingulum because other characters preponderantly distinguish the genus *Cherokia* from *Brachoria* (e.g., linear gonopods, the presence of a gonopodal coxal sternum in *Cherokia*, a 90°articulation between the telopodite and coxa in *Cherokia*, and others). The tibiotarsus, the indistinguishable fusion of the walking leg podomeres distal to the prefemur, which includes the basal zone, acropodite, apex and distal zone, is the most homoplasious region on the gonopods examined (Fig. 4). This is likely due to increased selective pressure on the region of the gonopod, which is physically inserted into the cyphopods during copulation (Tanabe & Sota, 2008). Whether selection is driven by female choice/SS, lock-and-key or some combination of the two is unknown. The high degree of attention that has traditionally been paid to tibiotarsal characters has led to a preponderance of these characters in morphological analyses, which undoubtedly biased our measures of region-specific homoplasy. However, the focus on tibiotarsal characters is due to the high degree of modification and variation of these features between species, relative to somatic characters (Shelley & Whitehead, 1986). These are salient features and immediately standout to the millipede taxonomist when examining material. Selection pressure under the female choice (SS) hypothesis leads to rapid phenotypic divergence, which can have the effect of increasing variation between species, or leading to morphological similarity due to convergence (Bond & Stockman, 2008; Eberhard, 1985). Conversely, the more-basally located coxa and prefemoral process may have a less active role in mating, such as serving to stabilize the tibiotarsus during copulation and/or to act as a receptacle for sperm collection, as in the distantly related Parajulidae (Mathews & Bultman, 1993). Such copulatory functions may receive a reduced degree of selection pressure, and our homoplasy analysis seems to support this (coxa + prefemur accounts for only 12% of gonopodal homoplasy, Fig. 4). This does not explain the relatively high degree of variation observed in the prefemoral processes of the Xystodesmidae, and the role of the prefemur during copulation warrants further study—particularly as it extends to the apex of the tibiotarsus in many taxa (e.g., Rhysodesmini, Nannariini, and *Riukiaria*) and appears to act in tandem with it (Tanabe & Sota, 2008).

### Systematic implications

**Figure 5:**
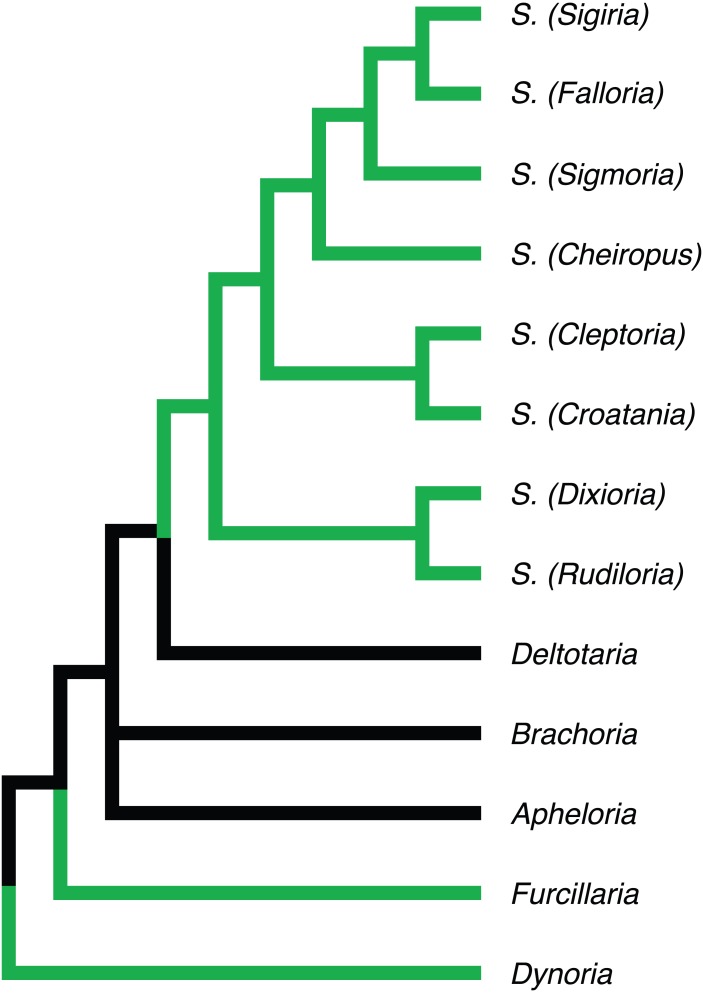
Phylogenetic relationships of Apheloriini genera as proposed by Shelley & Whitehead (1986), based on a combination of morphological and geographic data. Genera recovered as belonging to the “sigmoid” clade denoted with green branches.

The analysis presented here provides a basis to refine the previous classifications of Shelley & Whitehead (1986), Marek & Bond (2006) and Marek & Bond (2007) thereby allowing for revisionary syntheses of individual taxa and the description of new species (Fig. 5). In particular, genera that should be prioritized to receive a modern integrative revision, since they include a substantial number of new species, include *Sigmoria*, *Rudiloria*, *Apheloria*, and *Appalachioria*. For example, albeit not included in this analysis, the eastern US xystodesmid genus *Nannaria,* which is composed of 22 described species (tribe Nannariini and likely closely related to the Rhysodesmini, Marek & Bond, 2006), contains an estimated 200 species according to Hoffman (1964). However, based on unpublished revisionary work on the taxon by JCM, the estimated number of new species is more like 80. Still the number of undescribed species, and lack of an understanding of species relationships in the genus, is surprising and the taxon is among the least known alpha-taxonomically of all US fauna.

*Rhysodesmini, Pachydesmini, and sisters of the Apheloriini.* The southern Appalachian species *Boraria stricta* is a sister-group of the Pachydesmini clade, and distantly related to the other species of *Boraria*. To update the classification according to phylogeny, this might require the resurrection of the genus name *Aporiaria* for *B. deturkiana* and *B. infesta* (Fig. 2), since *B. stricta* is the genus type. The movement of *B. stricta* into the Pachydesmini would confuse the autopomorphies that define the Pachydesmini (i.e., lack of a gonopodal coxal sterna, with a gonopodal telepodite—coxal angle of 180°, lack of conspicuous coloration, and the relatively high rigidity of the body (Marek & Bond, 2006; Shelley & McAllister, 2006). *Boraria stricta* is differentiated by the presence of a gonopodal coxal sternum, a gonopodal telepodite—coxal angle of 90°, and other unique autapomorphic features of the gonopods (Shelley et al., 2011; Marek & Bond, 2006). This potential change in classification requires a more detailed investigation of the genus *Boraria*, including sampling additional “rhysodesmine” species and *B. profuga* from Arkansas. The Rhysodesmini is a paraphyletic set (grade) of lineages with respect to the Apheloriini. A paraphyletic Rhysodesmini agrees with a previous molecular study (Marek & Bond, 2006) and an as-yet unpublished molecular study rooted with taxa from outside of the Xystodesmidae, including *Melaphe* from the Mediterranean Basin (PE Marek, JC Means, D Hennen, 2004–2017, unpublished data). Pachydesmini is monophyletic and within the paraphyletic Rhysodesmini. Pachydesmini is known only from the eastern US with two genera just west of the Mississippi, *Pachydesmus* and *Thrinaxoria* (Shelley & McAllister, 2006). Rhysodesmini encompasses numerous additional taxa not included in this analysis (ca. 100 spp.) and the tribe is known from El Salvador north to New Mexico—where it is the most diverse. The taxon occurs north to Texas, the US Coastal Plain, and Appalachian Mountains where fewer species occur. Notably, *B. stricta* and *B. infesta* have been introduced to New York and Connecticut likely as a result of association with nursery and ornamental plants (Shelley et al., 2011). The genus *Deltotaria* is diagnosed by a distinct coxal process (Causey, 1942), which is lacking in other Xystodesmidae, and appear as two separate lineages at the base of the Apheloriini. The taxon, especially *Deltotaria brimleii brimleii*, possesses gonopods that are only slightly arcuate and appear as a possible transitional state between the linear gonopods of Rhysodesmini and the circular- and sigmoid-shaped ones of the Apheloriini. Future analyses should include the species *Deltotaria lea* to understand this potentially important morphological transition and explore the hypothesis of gonopod curvature to accommodate lengthening of the tibiotarsus for use in copulation.

*Apheloriini.* The Apheloriini composes 116 species in 17 genera and includes species with highly conspicuous aposematic coloration (Figs. 1A, 1C–1E) and taxa with geographically variable Müllerian mimicry (Marek, Tanabe & Sierwald, 2014). The sigmoid-clade (referred to in part as the genus *Sigmoria* in Shelley & Whitehead, 1986) is a large apheloriine clade represented in our phylogeny by *Furcillaria*, *Dynoria*, *Falloria*, *Dixioria watauga*, *Dixioria wrighti*, *Prionogonus*, *Croatania*, *Brevigonus*, and *Sigmoria* (Fig. 2, taxa in green). In the two-part treatise on the genus *Sigmoria* Shelley (1981) and Shelley & Whitehead (1986) placed the genera above (excluding *Furcillaria* and *Dynoria*), as well as *Cheiropus*, *Cleptoria*, *Lyrranea*, *Rudiloria* and *Stelgipus,* as subgenera of *Sigmoria*. This classification was recast by Hoffman (1999) who raised all twelve subgenera to generic status, some for what Hoffman saw as legitimate generic merits (such as *Dixioria*), and others for the simple reason of avoiding the “nomenclatorial complication” which subgenera presents (Hoffman, 1999, pg. 293). The analysis presented here indicates that the sigmoid-clade is much more heterogeneous (in general gonopodal morphology) than expected. The sigmoid-clade even includes the genera *Dynoria* and *Furcillaria*, which Shelley & Whitehead (1986) and Hoffman (1999) both recognized as sister to the Apheloriini based on their linear divided gonopods, and in the case of *Dynoria* lack of a twisted acropodite. The twisted acropodites of *Furcillaria* and the other Apheloriini, according to Shelley & Whitehead (1986) indicated a close affiliation, whereas the untwisted gonopods of *Dynoria* were suggested to be a shared ancestral feature with the groups outside of Apheloriini: the Pachydesmini, Nannariini, and Rhysodesmini. The sigmoid-clade is monophyletic and the prior genus conceptions, except *Falloria*, are polyphyletic and do not follow the MOT phylogeny. Both of the genera *Rudiloria* and *Dixioria*, which were included as subgenera of *Sigmoria* in SW86 are represented in clades outside of the sigmoid-clade. Using the genus name *Sigmoria* for this heterogeneous taxon is consistent with the phylogenetic pattern recovered in the tree. However, only 21 of the 74 described species are included in our analysis and additional species of *Sigmoria* and related genera should be included in future studies.

In our phylogeny, the genus *Rudiloria* is recovered as paraphyletic with respect to *Apheloria* (including *S. whiteheadi*) (Fig. 2). *Appalachioria separanda separanda*, a species with a cingulum, is sister to several species of *Rudiloria* from Kentucky, West Virginia and Ohio (*pp* = 1). This makes *Rudiloria* the fourth xystodesmid genus that contains a species with a cingulum; the others are *Appalachioria*, *Brachoria*, *Gonoessa,* and an undescribed species of *Nannaria*. The cingulum is a highly variable character and evolved a number of times independently across the xystodesmid tree. Use of the feature as a diagnostic character in Xystodesmidae higher-level taxonomy, and in other polydesmidan taxa might be critically reexamined and tempered with other morphological aspects. Marek & Bond (2006) performed a Bayesian ancestral-state reconstruction of the cingulum and found that it was unlikely to have evolved in the common ancestor of the clade spanning *Brachoria* and *Appalachioria* and more likely evolved independently in both *Appalachioria* and *Brachoria* lineages. *Gonoessa* and *Nannaria* (tribes Rhysodesmini and Nannariini) were not included in this study, but it would seem even more unlikely that the cingulum evolved in the common ancestor of the Rhysodesmini, Nannariini and Apheloriini. What would drive such a feature to develop independently in four different lineages of xystodesmid millipedes remains to be determined. Perhaps the cingulum serves as a point of articulation, which eases the coupling of the gonopods and cyphopods. The function of this character deserves a greater level of attention and it has been found in additional polydesmidan genera including *Aponedyopus* Verhoeff, 1939; *Atlantodesmus* Hoffman, 2000; *Chamberlinius* Wang, 1956; *Geniculodesmus* Chen, Golovatch & Chang, 2008; *Haplogonosoma* Brölemann, 1916; *Tylopus* Jeekel, 1968; *Riukiupeltis* Verhoeff, 1939 and *Simplogonomorpha* Nguyen & Korsos, 2011.

*Appalachioria* and *Brachoria* were once a single genus and have been recently examined using molecular phylogenetics (Marek & Bond, 2006; Marek & Bond, 2007; Marek, 2010). Based on our phylogenetic analysis, which includes thorough species sampling of the taxa, the genera are individually strongly supported as monophyletic (each with *pp* = 1). The *separanda* and *eutypa* species groups of *Appalachioria* are not monophyletic, and their species may require elevation to species level pending a revision of the genus (*Appalachioria separanda hamata*, *A. separanda versicolor*, *A. separanda calcaria* = the *separanda* group and *Appalachioria eutypa eutypa*, and *Appalachioria eutypa ethotela* = the *eutypa* group). *Appalachioria separanda calcaria* and *A. separanda versicolor* form a monophyletic clade (*pp* = 1) that may constitute one species. The evidence for this combination is threefold: (1) genetically they are only slightly distinct (consensus tree uncorrected *p*-distance =0.55), (2) geographically they form a continuum with overlapping distributions along the Brush Mountain range in southwest Virginia, and (3) morphologically the tip of the acropodite varies from a slight, smooth curve in *A. separanda calcaria* in Blacksburg, Virginia (at the northwest end of the parapatric species) to a characteristic *A. separanda versicolor* “hook” as one moves southwest to Marion, Virginia (at the southwest end of the species ranges). Both *A. separanda versicolor* and *A. separanda calcaria* are extremely variable in color, and some populations have four distinct color morphs. *Appalachioria eutypa eutypa* forms a monophyletic clade with *A. turneri* (*pp* = 0.74), and is distinct from *A. eutypa ethotela*. The changes to the prior *Brachoria* phylogeny, driven by the inclusion of additional gene regions in this study, are as follows: *B. mendota* is moved to sister of *B. evides*, *B. cedra* is moved to sister of a clade composed of B. *hansonia, B. glendalea, B. initialis, B. guntermountainensis, and B. ochra* (i.e., the western Tennessee clade; Marek, 2010). The clade composed of *B. hendrixsoni*, *B. hubrichti*, and *B. enodicuma* (Valley and Ridge clade; Marek, 2010) is sister to the clade of *B. hoffmani*, *B. sheari*, *B. insolita*, *B. dentata*, and *B. blackmountainensis* (Cumberland Mountain Thrust Block; Marek, 2010).

**Figure 6:**
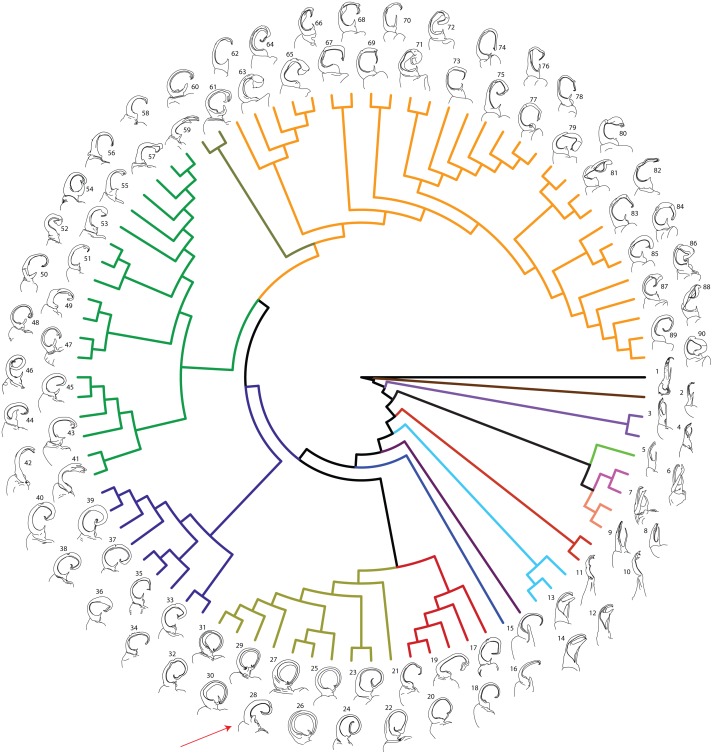
Molecular phylogeny of Xystodesmidae with left male gonopods (anterior view). Colors of branches as in Figs. 2 and 3. **1:** *Stenodesmus tuobitus*; **2:** *Gyalostethus monticolens*; **3:** *Boraria deturkiana*; **4:** *B. infesta*; **5:** *B. stricta*; **6:** *Pachydesmus crassicutis laticollis*; **7:** *P. c. incursus*; **8:***Dicellarius bimaculatus fictus*; **9:** *D. atlanta*; **10:** *Cherokia georgiana latassa*; **11:** *C. g. georgiana*; **12:** *Pleuroloma cala*; **13:** *P. plana*; **14:** *P. flavipes*; **15:** *Deltotaria brimleii philia*; **16:** *D. b. brimleii*; **17:*** Appalachioria separanda separanda*; **18:** *Rudiloria* n. sp. ‘Chagrin’; **19:** *R. guyandotta*; **20:** *R. mohicana*; **21:** *R. rigida*; **22:** *R. kleinpeteri*; **23:** *R. trimaculata trimaculata*; **24:** *Rudiloria* n. sp. ‘Monongahela’; **25:** *A. virginiensis reducta*; **26:** *A. v. corrugata*; **27:** *A. v. butleriana*; **28:** *Sigmoria whiteheadi*, indicated by a red arrow; **29:** *A. tigana*; **30:** *A. montana*; **31:** *Apheloria* n. sp. ‘Stone’; **32:** *Appalachioria falcifera*; **33:** *A. separanda hamata*; **34:** *Appalachioria* n. sp. ‘Clinch Mountain’; **35:** *A. eutypa eutypa*; **36:** *A. turneri*; **37:** *A. e. ethotela*; **38:** *Appalachioria* n. sp. ‘Foster’; **39:** *A. s. versicolor*; **40:*** A. s. calcaria*; **41:** *Furcillaria laminata*; **42:** *Dynoria medialis*; **43:** *Sigmoria nantahalae*; **44:** *Falloria prolata*; **45:** *F. xerophylla*; **46:** *F. aphelorioides*; **47**: *Dixioria watauga*; **48:** *D. wrighti*; **49:** *Sigmoria latior latior*; **50:** *S. l. hoffmani*; **51:** *S. nigrimontis*; **52:** *Prionogonus divergens*; **53:** *S. austrimontis*; **54:** *Croatania catawba*; **55:***S. australis*; **56:** *Brevigonus arcuatus*; **57:** *Brevigonus* n. sp. ‘Hagoods’; **58:** *B. shelfordi*; **59:** *Furcillaria aequalis*; **60:** *Dixioria coronata*; **61:** *D. dactylifera*; **62:** *Brachoria conta*; **63:** *B. viridicolens*; **64:** *B. plecta*; **65:** *B. calceata*; **66:** *B. indianae*; **67:** *B. divicuma*; **68:** *B. electa*; **69:** *B. evides*; **70:** *B. mendota*; **71:** *B. laminata*; **72:** *B. ligula*; **73:** *B. cedra*; **74:** *B. hansonia*; **75:** *B. glendalea*; **76:** *B. initialis*; **77:** *B. guntermountaineisis*; **78:** *B. ochra*; **79:** *B. splendida*; **80:** *B. kentuckiana*; **81:** *B. badbranchensis*; **82:** *B. gracilipes*; **83:** *B. hendrixsoni*; **84:** *B. hubrichti*; **85:** *B. enodicuma*; **86:** *B. hoffmani*; **87:** *B. sheari*; **88:** *B. insolita*; **89:** *B. dentata*; **90:** *B. blackmountaneisis*.

The genera *Rudiloria* and *Apheloria* are distinguished based on the tightly circular-shaped gonopods of *Apheloria* and the looser ovoid-shaped gonopods of *Rudiloria*. Notably, the genera have historically been united under the older name *Apheloria* by Chamberlin (1921) and Shear (1972). *Rudiloria mohicana*, *R. kleinpeteri* and *R. trimaculata trimaculata* have gonopods with fairly simple, circular acropodites (Fig. 6, #s 20, 22 and 23, respectively) and were at one point within the *Apheloria* due to this feature (Marek, Tanabe & Sierwald, 2014). Hoffman (1978) moved both species into *Rudiloria*, simply because they did not have quite as circular gonopods as the rest of *Apheloria*. This ovoid appearance of *Rudiloria* appears to be a shared, ancestral feature. Shear (1972) considered the morphological differences between *Apheloria* and *Rudiloria* to be of specific value only, and based on the phylogeny here and a pending revision of the genus *Apheloria*, *Rudiloria* should perhaps be synonymized with the older name *Apheloria*. Shelley & Whitehead (1986) suggested that *Rudiloria* is a subgenus of *Sigmoria*, and that a close affiliation between *Apheloria* and *Rudiloria* was unlikely, but with discovery of new species of *Rudiloria* from Monongahela National Forest in West Virginia, the similarity in gonopodal form is clearer and the pattern suggests that the tight circularity of *Apheloria* gonopods are derived from the ovoid-shaped gonopods of *Rudiloria*. Notably, both *Apheloria* and *Rudiloria* are part of Müllerian mimicry rings where they are codistributed in Appalachia with species of *Brachoria.* Mimicry between *Apheloria* and *Brachoria* is well known and has been documented, with some *Apheloria* species in a single locality possessing up to three distinct color morphs (Marek & Bond, 2009); however mimicry involving *Rudiloria* and these genera remains unstudied.

**Figure 7:**
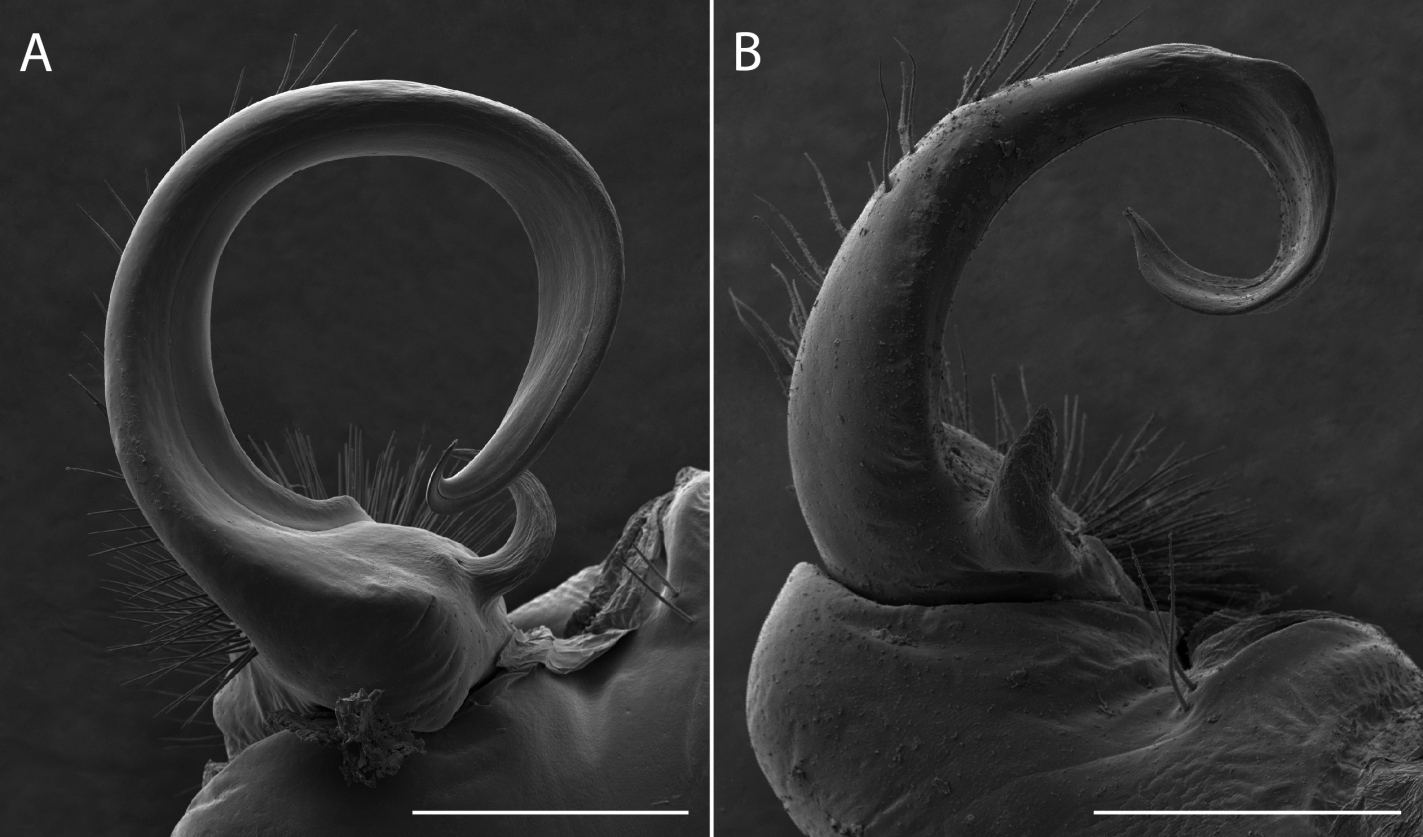
Scanning electron micrographs of the gonopods of *Apheloria virginiensis corrugata* (A) and *Sigmoria whiteheadi* (B), lateral view. Scale bar indicates 500 nm.

*Sigmoria whiteheadi* occurs within the *Apheloria* species clade (*pp* = 0.84 concatenated tree; and *pp* > 0.95 in the CO1 and 16S independent gene trees; Appendix D). This affiliation, based on cursory examination of the gonopods was unexpected due to its robust, sigmoid-shaped acropodite, stout, blunt prefemoral process and the presence of apical and medial flanges (Fig. 7). However, it is phylogenetically and geographically closer to *Apheloria* than the *rubromarginata*-group of *Sigmoria* that it was hypothesized to be closely related to by Shelley & Whitehead (1986), which occurs in the Southern Appalachians. Its highly contrasted lemon-yellow and black coloration is similar to *A. virginiensis corrugata* and *S. whiteheadi* even has traces of red coloration on the paranota—a hallmark of *A. virginiensis corrugata*. Based on this strong phylogenetic relationship and supported by a reexamination of morphology, the species is more closely related to *Apheloria* than it is to *Sigmoria* species. Therefore, we transfer *S. whiteheadi* into *Apheloria* and make the **new combination**, *Apheloria whiteheadi* (Shelley, 1986).

As indicated by a survey of the species to determine its geographical distribution, *A. whiteheadi* has an extremely restricted area (∼0.95 km^2^) and therefore may represent a relict or paleoendemic species that has significantly diverged from the rest of *Apheloria*. Whether *A. whiteheadi* was once more widespread and its range has contracted remains uncertain, but it certainly represents one of most geographically isolated species in the Apheloriini. Low vagility in combination with a strict reliance on stable mesic environments and a likely early evolutionary divergence has given rise to a number of short-range endemic species within the Xystodesmidae. A short-range endemic species (SRE) is defined as having a range of less than 10,000 km^2^ (Harvey et al., 2011; Harvey, 2002). Based on our sampling strategy, *A. whiteheadi* is an example of such a species, which is known from one location that is less than 1 km^2^ in area (Fig. 1A). The nine localities where *A. whiteheadi* were found form what appears to be a contiguous population, though the most northern location, near the headwaters of the Laurel Creek, is divided from the other localities by the Blue Ridge Parkway and a pasture, and may now represent a separated population.

Extreme short-range endemics such as *A. whiteheadi* are highly threatened by habitat fragmentation and environmental change and require careful study and conservation efforts to ensure their protection and the preservation of the unique habitat in which they persist. Worldwide, the Diplopoda are characterized by endemism and extremely restricted distributions (Enghoff, 2015) and the highly diverse Appalachian diplopod fauna are no exception. This is especially true in the Apheloriini, for example, 31 (91%) of the 34 species of *Brachoria* are SREs. Of the 90 taxa included in our study, 64 (71%) qualify as SREs, and 40 (44%) have distributions less than 1,000 km^2^, a threshold which Eberhard et al. (2009) suggests is more appropriate than the 10,000 km^2^ proposed by Harvey (2002). We agree with the original definition of an SRE by Harvey (2002) and propose the term Micro Range Endemic (MRE) for species with ranges less than 1,000 km^2^.

A consideration when designating a species as an SRE or MRE is the method by which the distributional area is measured, especially if these distribution data are used for conservation efforts or governmental regulation (Harvey et al., 2011). For the taxa in this study we georeferenced collection records from literature (see Methods), input them into ArcGIS (ESRI, Redlands, CA, USA) and drew a polygon connecting the peripheral collection sites. From this polygon we could measure the area, however the drawing of the polygon may be inherently subject to bias. By drawing a polygon connecting collection sites, the suitability of the habitat included in the polygon is ignored, which may further over-estimate species distribution areas. Alternatively, species distributions may be underestimated through a lack of sampling, which further reinforces the need for collection efforts in the Appalachians and other under sampled, remote regions of the world to gather these critical data (Harvey et al., 2011).

SRE and MRE taxa present complex challenges for conservation efforts (Harvey et al., 2011; Eberhard et al., 2009; Harvey, 2002). In the case of the millipedes, genera with multiple SRE and MRE taxa, such as the *Brachoria* and *Sigmoria*, form a contiguous mosaic-like patchwork across the Appalachians, and the protection of each one of these small habitats may be challenging. Additionally, SRE and especially MRE species are highly sensitive to environmental alteration, and the legal protection of habitat itself (thereby reducing direct threats via land-use change) may not be sufficient to mitigate the broader effects of air and water pollution and global climate change. Rather than be discouraging, these challenges should inspire conservation groups to work creatively to protect these unique, and highly threatened species.

## Conclusion and Future Directions

The homoplasy found in our morphological tree was high, and raises concerns about the utility of analyses reconstructing relationships above the species level in millipedes based solely on morphological data. Our comparison of phylogenetic hypotheses demonstrated a high level of discordance between the various methods that taxonomists used to assess evolutionary relationships in the millipede family Xystodesmidae. While the historical phylogeny (SW86) was the most consistent with our multi-gene molecular phylogeny, the tree was still highly divergent. Morphology and geography appear to be useful in combination for taxon delimitation at the genus level, but when considered separately as in the independent EDT and MRT trees there is a substantial reduction in accuracy. We showed that the majority of gonopodal features have high homoplasy as a result of convergence and are not useful for taxon delimitation above the genus-level, but when used at the species level are effective as species-characteristic features. In the field, geography with morphology is a good combination for quick taxa identification. While molecular phylogenetics is also affected by rampant convergence, negative effects are mitigated through the presence of several orders of magnitude more characters than morphological analyses and by combining loci and assessing concordance among gene trees.

The molecular phylogeny presented here re-examines some of the relationships within the Xystodesmidae. More detailed examination of the genera and tribes within the Xystodesmidae is warranted. While our study included 90 species in 20 genera, there are 393 species and 62 genera in Xystodesmidae. This includes unsampled members from the Russian Far East, the Mediterranean Basin, and Ethiopia. Moreover, there are ca. 70 new species of the genus *Nannaria* in the eastern US and ca. 10 new species of Xystodesmidae have been described from the mountains of southwest China and Vietnam over the past several years. Future taxonomic treatments should include an increased taxon sampling to enhance accuracy and to not miss cryptic systematic relationships as shown with *A. whiteheadi* and *Apheloria*. The family has a high diversity in Appalachia within small geographical areas, indicating an unparalleled and irreplaceable diversity that is threatened by any level of habitat loss. We hope that this study will spur future work on Xystodesmidae, and will provide an introductory basis for those interested in exploring this fascinating group of organisms.

##  Supplemental Information

10.7717/peerj.3854/supp-1 Appendix A List of taxa used in both the morphological and molecular analyses List of taxa used in both the morphological and molecular analyses, organized alphabetically by genus and then species. *Taxa that were represented by separate specimens in the morphological and molecular analyses, molecular specimen code shown, morphological specimen as in Marek & Bond (2006) (Appendix A). Localities refer to male specimens only. All specimens available from the corresponding author by request and stored in the Virginia Tech Insect Collection, Blacksburg, Virginia, USA.

10.7717/peerj.3854/supp-2 Appendix B Binary and multistate characters used for scoring of morphological matrix DT: dwell time spent in each character state; HI: homoplasy index. Due to the lack of female specimens and heads in some taxa the female (41–45) and cephalic (46–47) characters were not included in the stochastic character state analysis.

10.7717/peerj.3854/supp-3 Appendix C DNA Amplification Procedures DNA amplification procedures for the six genes used in this study: COI; EF1a; tRNA-VAL, 12S and 16S (denoted as 12S–16S in text); and 28S.

10.7717/peerj.3854/supp-4 Appendix D Independent gene trees for: COI, EF1a, 16S/12S/tRNA-Val and 28S Independent gene trees for the six genes used in this study. Turquoise bars indicate sit substitution rates and asterisks indicate posterior probability node support >0.70.

10.7717/peerj.3854/supp-5 Supplemental Information 1 Morphology scoring matrix

10.7717/peerj.3854/supp-6 Supplemental Information 2 Species distribution perimeters

## Additional Information and Declarations

### Field Study Permissions

The following information was supplied relating to field study approvals (i.e., approving body and any reference numbers): The United States Department of the Interior National Park Service (Permit #: BLRI-2014-SCI-0033) approved this study.

### DNA Deposition

The following information was supplied regarding the deposition of DNA sequences: The aligned sequences used in the phylogenetic analysis have been deposited at GenBank. For a complete list of the sequences and the corresponding accession numbers, see Appendix A.

### Data Availability

The following information was supplied regarding data availability: Repository: VTechData
https://data.lib.vt.edu/collections/wd375w32h. doi: 10.7294/W4GM85G8. This raw data has also been provided as Supplemental Files.
