# Supplementary material for: Is geography an accurate predictor of evolutionary history in the millipede family Xystodesmidae?
Source: PeerJ. 2017 Oct 12;5:e3854. doi: 10.7717/peerj.3854 (PMC5641431; doi:10.7717/peerj.3854)
Supplement: Appendix A — List of taxa used in both the morphological and molecular analyses, organized alphabetically by genus and then species. *Taxa that were represented by separate specimens in the morphological and molecular analyses, molecular specimen code shown, morphological specimen as in Marek & Bond (2006) (Appendix A). Localities refer to male specimens only. All specimens available from the corresponding author by request and stored in the Virginia Tech Insect Collection, Blacksburg, Virginia, USA. [file peerj-05-3854-s001.doc]

**Appendix A**

| **Taxon** | **Spc# - M** | **Spc# - F** | **Latitude** | **Longitude** | **State, County** | **Acc# COI** | **Acc# EF1a** | **Acc# 16S** | **Acc# 28S** |
| --- | --- | --- | --- | --- | --- | --- | --- | --- | --- |
| **Apheloriini**  *Apheloria montana* (Bollman, 1888) | SPC000134 | SPC000133 | 35.73446 | -82.08378 | North Carolina, McDowell | **KR135989** | **KR136042** | **DQ490660** | **KR135887** |
| *Apheloria* n. sp. ‘Monongahela’ | SPC000717 | SPC000729 | 38.27285 | -80.52504 | West Virginia, Nicholas | **MF953749** | **MF953819** | **EU127865** | **MF948869** |
| *Apheloria* n. sp. ‘Stone’ | SPC000578 | - | 36.75603 | -83.19588 | Kentucky, Harlan | **MF953743** | **MF953815** | **EU127857** | **MF948866** |
| *A. tigana* Chamberlin, 1939 | SPC000311 | - | 35.84460 | -78.75750 | North Carolina, Wake | **MF953721** | **MF953798** | **DQ490687** | **MF948846** |
| *A. virginiensis butleriana* (Bollman, 1889) | SPC000997 | - | 38.72190 | -85.46120 | Indiana, Madison | **MF953754** | **MF953823** | **MF953677** | **MF948874** |
| *A. v. corrugata* (Wood, 1864) | MPE00745 | MPE00766 | 37.02528 | -80.77522 | Virginia, Pulaski | **MF953686** | **MF953769** | **MF953667** | **-** |
| *A. v. reducta* Chamberlin, 1939 | MPE00001 | - | 36.44294 | -93.75115 | Arkansas, Carroll | **MF953680** | **MF953763** | **MF953661** | **-** |
| *Appalachioria eutypa ethotela* (Chamberlin, 1942) | SPC000293 | SPC000291 | 36.88190 | -81.52340 | Virginia, Smyth | **MF953719** | **MF953796** | **DQ490685** | **MF948844** |
| *A. eutypa eutypa* (Chamberlin, 1939) | SPC000226 | SPC000229 | 36.07860 | -81.77860 | North Carolina, Avery | **MF953706** | **MF953787** | **DQ490668** | **MF948831** |
| *A. falcifera* (Keeton, 1959) | SPC000259 | - | 37.12882 | -81.87666 | Virginia, Tazewell | **MF953713** | **MF953791** | **DQ490677** | **MF948838** |
| *Appalachioria* n. sp. ‘Clinch Mountain’ | SPC000282 | - | 36.72317 | -82.29852 | Virginia, Washington | **MF953717** | **MF953794** | **DQ490682** | **MF948842** |
| *Appalachioria* n. sp. ‘Foster’ | SPC000296 | SPC000294 | 36.89021 | -80.83755 | Virginia, Wythe | **MF953720** | **MF953797** | **DQ490686** | **MF948845** |
| *A. separanda calcaria* (Keeton, 1959) | SPC000516 | - | 37.22360 | -80.38690 | Virginia, Montgomery | **MF953738** | **MF953811** | **EU127852** | **MF948861** |
| *A. s. hamata* (Keeton, 1959) | SPC000325 | - | 37.08271 | -81.30132 | Virginia, Tazewell | **MF953723** | **MF953799** | **DQ490689** | **MF948848** |
| *A. s. separanda* (Chamberlin, 1947) | SPC000511 | - | 39.54947 | -79.14581 | Maryland, Garrett | **MF953737** | **-** | **MF953673** | **MF948860** |
| *A. s. versicolor* (Hoffman, 1963) | MPE00643 | MPE01193 | 37.02230 | -81.20483 | Virginia, Wythe | **MF953684** | **MF953767** | **MF953665** | **MF948810** |
| *A. turneri* (Keeton, 1959) | SPC000288 | SPC000289 | 36.81711 | -81.92088 | Virginia, Washington | **MF953718** | **MF953795** | **DQ490684** | **MF948843** |
| *Brachoria badbranchensis* Marek, 2010 | SPC001047 | - | 37.08020 | -82.77232 | Kentucky, Letcher | **MF953760** | **-** | **EU127873** | **MF948879** |
| *B. blackmountainensis* Marek, 2010 | SPC001039 | - | 36.94995 | -82.87520 | Kentucky, Harlan | **MF953758** | **MF953827** | **EU127872** | **-** |
| *B. calceata* (Causey, 1955) | SPC000625 | SPC000628 | 38.01506 | -84.82410 | Kentucky, Woodford | **MF953747** | **-** | **EU127861** | **-** |
| *B. cedra* Keeton, 1959 | SPC000276 | - | 36.66032 | -83.18390 | Virginia, Lee | **MF953716** | **MF953793** | **DQ490680** | **MF948841** |
| *B. conta* Keeton, 1965 | SPC000640 | SPC000642 | 38.33792 | -83.05760 | Kentucky, Carter | **MF953748** | **MF953818** | **EU127862** | **-** |
| *B. dentata* Keeton, 1959* | SPC000536 | - | 36.77681 | -83.01574 | Virginia, Lee | **MF953740** | **MF953813** | **EU127854** | **MF948863** |
| *B. divicuma* Keeton, 1965 | SPC000453 | SPC000458 | 36.44180 | -84.85820 | Tennessee, Fentress | **MF953733** | **MF953807** | **EU127847** | **MF948857** |
| *B. electa* Causey, 1955* | SPC000613 | - | 38.02163 | -84.83765 | Kentucky, Anderson | **MF953746** | **-** | **EU127860** | **MF948868** |
| *B. enodicuma* Keeton, 1965* | SPC000467 | - | 34.64966 | -85.94576 | Alabama, Jackson | **MF953735** | **MF953809** | **EU127849** | **MF948858** |
| *B. evides* (Bollman, 1888) | SPC000440 | SPC000442 | 36.21869 | -83.16277 | Tennessee, Hamblen | **MF953732** | **MF953806** | **EU127846** | **MF948856** |
| *B. glendalea* (Chamberlin, 1918)* | SPC000481 | - | 36.09468 | -86.79347 | Tennessee, Davidson | **MF953736** | **MF953810** | **EU127850** | **MF948859** |
| *B. gracilipes* (Chamberlin, 1947) | SPC001084 | - | 36.93504 | -83.20281 | Kentucky, Harlan | **MF953761** | **MF953829** | **GU150156** | **MF948880** |
| *B.* *guntermountainensis* Marek. 2010 | SPC000071 | SPC000070 | 34.60658 | -86.11101 | Alabama, Jackson | **MF953693** | **-** | **DQ490653** | **MF948816** |
| *B. hansonia* Causey, 1950 | SPC000554 | SPC000343 | 36.71301 | -83.73531 | Kentucky, Ball | **MF953741** | **MF953814** | **EU127855** | **MF948864** |
| *B.* *hendrixsoni* Marek, 2010 | SPC000220 | SPC000217 | 36.97738 | -82.84390 | Tennessee, Greene | **MF953703** | **MF953786** | **DQ490666** | **MF948828** |
| *B. hoffmani* Keeton, 1959 | SPC000261 | SPC000265 | 37.29292 | -82.30893 | Virginia, Dickenson | **MF953714** | **MF953792** | **DQ490678** | **MF948839** |
| *B. hubrichti* Keeton, 1959* | SPC000460 | - | 35.09220 | -85.64670 | Tennessee, Marion | **MF953734** | **MF953808** | **EU127848** | **-** |
| *B. indianae* (Bollman, 1888)* | SPC000993 | - | 38.48170 | -85.50980 | Indiana, Clark | **MF953753** | **MF953822** | **EU127867** | **MF948873** |
| *B. initialis* Chamberlin, 1939 | SPC000083 | SPC000084 | 31.73469 | -88.19488 | Alabama, Choctaw | **-** | **MF953779** | **DQ490658** | **MF948821** |
| *B. insolita* Keeton, 1959 | SPC000275 | - | 36.89532 | -82.60513 | Virginia, Wise | **MF953715** | **-** | **DQ490679** | **MF948840** |
| *B. kentuckiana* (Causey, 1942) | SPC000599 | SPC000603 | 36.84121 | -84.34126 | Kentucky, Whitely | **MF953744** | **MF953816** | **EU127858** | **MF948867** |
| *B. laminata* Keeton, 1959 | SPC000258 | SPC000264 | 37.16602 | -81.70370 | Virginia, Tazewell | **MF953712** | **-** | **DQ490676** | **MF948837** |
| *B. ligula* Keeton, 1959 | SPC000324 | SPC000322 | 37.43356 | -81.57610 | West Virginia, McDowell | **MF953722** | **-** | **DQ490688** | **MF948847** |
| *B. mendota* Keeton, 1959* | SPC000525 | - | 36.72868 | -82.30478 | Virginia, Russell | **MF953739** | **MF953812** | **EU127853** | **MF948862** |
| *B. ochra* (Chamberlin, 1918) | SPC000077 | SPC000091 | 34.30959 | -87.39433 | Alabama, Lawrence | **MF953695** | **-** | **DQ490655** | **MF948818** |
| *B. plecta* Keeton, 1959 | SPC000561 | SPC000636 | 37.77415 | -83.68987 | Kentucky, Powell | **MF953742** | **-** | **EU127856** | **MF948865** |
| *B. sheari* Marek, 2010 | SPC001030 | - | 36.60764 | -83.62952 | Virginia, Lee | **MF953757** | **MF953826** | **EU127871** | **MF948877** |
| *B. splendida* (Causey, 1942) | SPC000341 | SPC000344 | 36.73537 | -83.73924 | Kentucky, Bell | **MF953725** | **MF953801** | **DQ490693** | **MF948850** |
| *B. viridicolens* (Hoffman, 1948) | SPC000606 | SPC000611 | 37.27314 | -85.49036 | Kentucky, Green | **MF953745** | **MF953817** | **EU127859** | **MF962576** |
| *Brevigonus arcuatus* Shelley, 1981 | SPC000423 | SPC000420 | 34.40480 | -82.57786 | South Carolina, Anderson | **MF953731** | **-** | **DQ490699** | **MF948855** |
| *Brevigonus* n. sp. ‘Hagoods’ | SPC000389 | SPC000390 | 33.21206 | -81.31976 | South Carolina, Barnwell | **MF953729** | **-** | **DQ490697** | **MF948853** |
| *B. shelfordi* Loomis, 1944* | SPC001130 | - | 33.93267 | -82.38731 | South Carolina, Abbeville | **MF953762** | **MF953830** | **MF953679** | **MF948881** |
| *Croatania catawba* (Shelley, 1977)* | SPC001015 | - | 35.21320 | -81.29370 | North Carolina, Gaston | **MF953756** | **MF953825** | **EU127869** | **MF948876** |
| *Deltotaria brimleii brimleii* Causey, 1942 | SPC000142 | - | 35.63648 | -83.49181 | Tennessee, Sevier | **MF953699** | **MF953782** | **DQ490662** | **MF948824** |
| *D. brimleii philia* (Chamberlin, 1949) | SPC000047 | - | 34.80362 | -83.12994 | South Carolina, Oconee | **MF953691** | **MF953775** | **DQ490651** | **-** |
| *Dixioria coronata* (Hoffman, 1949) | SPC000166 | SPC000163 | 36.65547 | -81.58620 | Virginia, Smyth | **MF953702** | **MF953785** | **DQ490665** | **MF948827** |
| *D. dactylifera* (Hoffman, 1956) | SPC000223 | - | 36.40880 | -81.58610 | North Carolina, Ashe | **MF953704** | **-** | **DQ490667** | **MF948829** |
| *D. watauga* Shelley, 1986 | MPE00325 | MPE00309 | 36.20490 | -81.72920 | North Carolina, Watauga | **MF953681** | **MF953764** | **MF953661** | **MF948808** |
| *D. wrighti* Hoffman, 1956 | SPC000828 | SPC000829 | 36.08310 | -81.77680 | North Carolina, Avery | **MF953751** | **-** | **MF953675** | **MF948871** |
| *Dynoria medialis* Chamberlin, 1949 | SPC000431 | SPC000427 | 33.25058 | -83.92334 | Georgia, Butts | **KR135993** | **KR136045** | **DQ490700** | **KR135891** |
| *Falloria aphelorioides* Shelley, 1986 | SPC000907 | SPC000910 | 35.42604 | -84.06647 | Tennessee, Monroe | **MF953752** | **MF953821** | **MF953676** | **MF948872** |
| *F. prolata* Shelley, 1986 | SPC000145 | SPC000147 | 35.72054 | -83.39545 | Tennessee, Sevier | **MF953700** | **MF953783** | **DQ490663** | **MF948825** |
| *F. xerophylla* (Shelley, 1981) | SPC000055 | - | 34.75646 | -84.70615 | Georgia, Murray | **MF953692** | **MF953776** | **DQ490652** | **MF948815** |
| *Furcillaria aequalis* Shelley, 1981* | SPC001132 | - | 33.72940 | -82.18419 | South Carolina, McCormick | **MF959749** | **MF959748** | **MF959747** | **MF962577** |
| *Furcillaria laminata* Shelley, 1981 | SPC000421 | SPC000424 | 34.40480 | -82.57786 | South Carolina, Anderson | **MF953730** | **MF953805** | **DQ490698** | **MF948854** |
| *Prionogonus divergens* Chamberlin, 1939 | SPC000039 | SPC000041 | 35.11707 | -82.63942 | South Carolina, Greenville | **MF953690** | **MF953774** | **DQ490650** | **MF948814** |
| *Rudiloria guyandotta* (Shear, 1972) | SPC001043 | SPC001045 | 37.03520 | -82.96350 | Kentucky, Letcher | **MF953759** | **MF953828** | **MF953678** | **MF9I have48878** |
| *R. kleinpeteri* (Hoffman, 1949) | SPC000164 | - | 36.71343 | -81.46003 | Virginia, Grayson | **MF953701** | **MF953784** | **DQ490664** | **MF948826** |
| *R. mohicana* (Causey, 1955)* | MPE00496 | - | 39.34265 | -82.01758 | Ohio, Athens | **MF953682** | **MF953765** | **MF953663** | **-** |
| *Rudiloria* n. sp. ‘Chagrin’ | MTX0185 | - | 41.41598 | -81.41448 | Ohio, Cuyahoga | **MF953687** | **MF953770** | **MF953668** | **MF948811** |
| *R. rigida* Shelley, 1986 | SPC000722 | SPC000728 | 38.27285 | -80.52504 | West Virginia, Nicholas | **MF953750** | **MF953820** | **MF953674** | **MF948870** |
| *R. trimaculata trimaculata* (Wood, 1864) | SPC000253 | SPC000252 | 37.42802 | -80.49935 | Virginia, Giles | **MF953711** | **-** | **DQ490674** | **MF948836** |
| *Sigmoria australis* Shelley, 1986 | SPC000080 | SPC000082 | 30.57910 | -84.94170 | Florida, Liberty | **MF953697** | **MF953778** | **DQ490657** | **MF948820** |
| *S. austrimontis* Shelley, 1981 | SPC000224 | SPC000132 | 35.66050 | -81.69800 | North Carolina, Burke | **MF953705** | **-** | **MF953671** | **MF948830** |
| *S. latior hoffmani* Shelley, 1976 | SPC000368 | SPC000372 | 33.28397 | -79.61884 | South Carolina, Berkeley | **MF953727** | **MF953803** | **MF953672** | **-** |
| *S. latior latior* (Brölemann, 1900)* | SPC000227 | - | 36.07860 | -81.77860 | North Carolina, Avery | **MF953707** | **MF953788** | **DQ490669** | **MF948832** |
| *S. nantahalae* Hoffman, 1958 | SPC000244 | SPC000249 | 35.34880 | -83.97680 | North Carolina, Graham | **MF953708** | **MF953789** | **DQ490670** | **MF948833** |
| *S. nigrimontis* (Chamberlin, 1947)* | SPC000246 | - | 35.76480 | -82.26510 | North Carolina, Yancey | **MF953709** | **-** | **DQ490671** | **MF948834** |
| *S. whiteheadi* Shelley, 1986 | MPE00712 | MPE00447 | 36.77245 | -80.40544 | Virginia, Floyd | **MF953685** | **MF953768** | **MF953666** | **-** |
|  |  |  |  |  |  |  |  |  |  |
| **Rhysodesmini**  *Boraria deuturkiana* (Causey, 1942) | MPE00504 | MPE01479 | 35.08157 | -83.23599 | North Carolina, Macon | **MF953683** | **MF953766** | **MF953664** | **MF948809** |
| *B. infesta* (Chamberlin, 1918) | SPC000248 | SPC000232 | 35.76480 | -82.26510 | North Carolina, Yancey | **MF953710** | **MF953790** | **DQ490672** | **MF948835** |
| *B. stricta* (Brölemann, 1896) | SPC000135 | SPC000241 | 35.83420 | -82.40938 | North Carolina, Yancey | **-** | **MF953781** | **DQ490661** | **MF948823** |
| *Cherokia georgiana georgiana* (Bollman, 1889) | SPC000354 | SPC000356 | 35.06337 | -83.43687 | North Carolina, Macon | **MF953726** | **MF953802** | **DQ490695** | **MF948851** |
| *C. georgiana latassa* Hoffman, 1960 | SPC000073 | SPC000072 | 34.60658 | -86.11101 | Alabama, Jackson | **MF953694** | **MF953777** | **DQ490654** | **MF948817** |
| *Gyalostethus monticolens* (Chamberlin, 1951) | SPC001008 | - | 36.76070 | -83.14000 | Kentucky, Harlan | **MF953755** | **MF953824** | **EU127868** | **MF948875** |
| *Pleuroloma cala* (Chamberlin, 1939) | MTX0221 | - | 29.15457 | -81.56577 | Florida, Lake | **MF953688** | **MF953772** | **MF953669** | **MF948812** |
| *P. flavipes* Rafinesque, 1820 | SPC000338 | SPC000340 | 36.92891 | -83.19141 | Kentucky, Bell | **MF953724** | **MF953800** | **DQ490692** | **MF948849** |
| *P. plana* Shelley, 1980 | SPC000119 | SPC000115 | 30.57910 | -84.94170 | Florida, Liberty | **MF953698** | **MF953780** | **DQ490659** | **MF948822** |
| *Stenodesmus tuobitus* (Chamberlin, 1910) | MTX0199 | - | 32.93085 | -108.0137 | New Mexico, Grant | **KR136021** | **MF953771** | **KR135967** | **KR135918** |
| **Pachydesmini**  *Dicellarius atlanta* (Chamberlin, 1946) | SPC000428 | A1855 | 33.25058 | -83.92334 | Georgia, Butts | **KR135992** | **KR136044** | **DQ490648** | **KR135890** |
| *D. bimaculatus fictus* (Chamberlin, 1943) | SPC000079 | - | 30.57910 | -84.94170 | Florida, Liberty | **MF953696** | **-** | **DQ490656** | **MF948819** |
| *Pachydesmus crassicutis incursus* Chamberlin, 1939 | SPC000380 | SPC000397 | 33.13690 | -81.43390 | South Carolina, Barnwell | **MF953728** | **MF953804** | **DQ490696** | **MF948852** |
| *P. crassicutis laticollis* (Attems, 1899) | MTX0419 | SPC000010 | 31.43665 | -90.98462 | Mississippi, Franklin | **MF953689** | **MF953773** | **MF953670** | **MF948813** |
|  |  |  |  |  |  |  |  |  |  |

List of taxa used in both the morphological and molecular analyses, organized alphabetically by genus and then species. *Taxa that were represented by separate specimens in the morphological and molecular analyses, molecular specimen code shown, morphological specimen as in Marek and Bond, 2006 (Appendix A). Localities refer to male specimens only. Acc# refer to the NCBI Genbank database. All specimens available from the corresponding author by request and stored in the Virginia Tech Insect Collection, Blacksburg, Virginia, USA
