# Supplementary material for: Is geography an accurate predictor of evolutionary history in the millipede family Xystodesmidae?
Source: PeerJ. 2017 Oct 12;5:e3854. doi: 10.7717/peerj.3854 (PMC5641431; doi:10.7717/peerj.3854)
Supplement: Appendix B — DT: dwell time spent in each character state; HI: homoplasy index. Due to the lack of female specimens and heads in some taxa the female (41–45) and cephalic (46–47) characters were not included in the stochastic character state analysis. [file peerj-05-3854-s002.docx]

**Appendix B**

Binary and multistate characters used for scoring of morphological matrix. DT: dwell time spent in each character state; HI: homoplasy index. Due to the lack of female specimens and heads in some taxa the female (41-45) and cephalic (46-47) characters were not included in the stochastic character state analysis.

1. ♂ Gonopodal coxal sternum, presence: present, coxae connected with sternum (0), DT = 0.4044; absent, coxae connected with membrane only (1), DT = 0.5956; HI = 0.7199.
2. ♂ Gonopodal coxal apophysis, presence: absent (0), DT = 0.9362; present (1), DT = 0.0638; HI = 0.8088.
3. ♂ Gonopodal telopodite-coxa angle: 90 degree articulation between telopodite and coxa (0), DT = 0.4950; 180 degree articulation between telopodite and coxa (1), DT = 0.9499; HI = 0.3671.
4. ♂ Gonopodal prefemoral process, presence: present (0), DT = 0.9499; absent (1), DT = 0.0501; HI = 0.9196.
5. ♂ Gonopodal prefemoral process, shape: present, long, acicular (0), DT = 0.4603; present, short, stout (1), DT = 0.4995; absent (2), DT = 0.0402; HI = 0.8358.
6. ♂ Gonopodal basal zone inner surface, orientation relative to a perpendicular axis arising ventrally from the gonopodal coxa: twisted anteromedially (0), DT = 0.5137; medially (1), DT = 0.4863; HI = 0.9751.
7. ♂ Gonopodal basal zone tubercle one, presence: absent (0), DT = 0.9815; present (1), DT = 0.0185; HI = 0.8387.
8. ♂ Gonopodal basal zone tubercle two, presence: absent (0), DT = 0.9834; present (1), DT = 0.0166; HI = 0.8792.
9. ♂ Gonopodal basal zone tubercle two, size: absent (0), DT = 0.9425; enlarged (1), DT = 0.0337; large spur (2), DT = 0.0238; HI = 0.9961.
10. ♂ Gonopodal basal zone medial flange, presence: absent (0), DT = 0.9243; present (1), DT = 0.0757; HI = 0.9601.
11. ♂ Gonopodal anterior bend + apical curve, presence: absent (0), DT = 0.5282; present (1), DT = 0.4718; HI = 0.8208.
12. ♂ Gonopodal basal and distal zones, planation: distal zone absent (0), DT = 0.5008; not coplanar (1), DT = 0.4749; coplanar, distal zone at a right angle from the peak (2), DT = 0.0243; HI = 0.8486.
13. ♂ Gonopodal anterior twist, definition: anterior twist absent (0), DT = 0.4703; broad and poorly defined, slightly twisted cephalically (1), DT = 0.3213; well-defined and strongly twisted cephalically (2), DT = 0.2084; HI = 0.9521.
14. ♂ Gonopodal "medial" flange, presence: absent (0), DT = 0.6474; present (1), DT = 0.3526; HI = 0.9946.
15. ♂ Gonopodal acropodite medial margin tooth, presence: absent (0), DT = 0.8718; present (1), DT = 0.1282; HI = 0.9519.
16. ♂ Gonopodal peak tooth, presence: absent (0), DT = 0.9342; present (1), DT = 0.0658; HI = 0.9429.
17. ♂ Gonopodal acropodite medial margin accessory tooth, presence: absent (0), DT = 0.9809; present (1), DT = 0.0191; HI = 0.7899.
18. ♂ Gonopodal distal zone-peak, angle: straight, 0 degrees (0), DT = 0.5166; bent, non-circular angle to 90 degrees (1), DT = 0.4393; circular (2), DT = 0.0441; HI = 0.8246.
19. ♂ Gonopodal distal zone, area distal to apical curve, orientation: distal zone absent, straight gonopod (0), DT = 0.4831; curved medially (1), DT = 0.4860; curved laterally (2), DT = 0.0309; HI = 0.8997.
20. ♂ Gonopodal distal zone, length: distal zone absent (0), DT = 0.2434; short, less than 0.4X length of acropodite (1), DT = 0.4299; long, greater than or equal to 0.5X length of acropodite (2), DT = 0.3267; HI = 0.9867.
21. ♂ Gonopodal lateral flange, presence: absent (0), DT = 0.5058; present (1), DT = 0.4942; HI = 0.9979.
22. ♂ Gonopodal lateral flange, orientation: absent (0), DT = 0.5174; anterolaterally (1), DT = 0.2650; anterodorsally (2), DT = 0.1310; dorsolaterally (3), DT = 0.0575; posterolaterally (4), DT = 0.0291; HI = 0.9937.
23. ♂ Gonopodal lateral flange, shape: absent (0), DT = 0.5200; laminate (1), DT = 0.2658; lobe-like (2), DT = 0.2141; HI = 0.9948.
24. ♂ Gonopodal acropodite distal zone surface, orientation relative to a perpendicular axis arising ventrally from the gonopodal coxa: prolaterally, linear acropodite (0), DT = 0.3717; anterolaterally, twisted 1/4 turn clockwise (1), DT = 0.3709; posterolaterally, twisted 1/4 turn counterclockwise (2), DT = 0.2212; ventrally, twisted 3/4 turn clockwise (3), DT = 0.0362; HI = 0.9436.
25. ♂ Gonopodal acropodite, expansion: tapering to a point distally (0), DT = 0.7113; constant width (1), DT = 0.1223; expanded distally (2), DT = 0.1665; HI = 0.9951.
26. ♂ Gonopodal additional apical process, presence: absent (0), DT = 0.6133; present (1), DT = 0.3867; HI = 0.9730.
27. ♂ Gonopodal solenomere (those with an additional apical process), position: absent (0), DT = 0.5487; medial (1), DT = 0.0686; anterolateral (2), DT = 0.1344; posterolateral (3), DT = 0.2484; HI = 0.9832.
28. ♂ Gonopodal fold, presence: absent (0), DT = 0.9637; present (1), DT = 0.0363; HI = 0.4118.
29. ♂ Gonopodal cingulum, presence: absent (0), DT = 0.7868; present (1), DT = 0.2132; HI = 0.8043.
30. ♂ Gonopodal cingulum, location: absent (0), DT = 0.7651; proximal (1), DT = 0.0689; distal (2), DT = 0.1660; HI = 0.8945.
31. ♂ Gonopodal acropodite anterior bend, presence: absent (0), DT = 0.3283; present (1), DT = 0.6717; HI = 0.7368.
32. ♂ Gonopodal acropodite apical curve, presence: absent (0), DT = 0.5273; present (1), DT = 0.4727; HI = 0.8066.
33. ♂ Gonopodal acropodite peak, percent of acropodite distal from peak: 0, straight acropodite (0), DT = 0.3437; 17-33 (1), DT = 0.2022; 40 (2), DT = 0.2528; 40-70 (3), DT = 0.2012; HI = 0.9793.
34. ♂ Gonopodal apical acropodite, percent divided: 0, not divided (0), DT = 0.6778; 16.6 (1), DT = 0.1047; 30 (2), DT = 0.1771; 100 (3), DT = 0.0405; HI = 0.9229.
35. ♂ Gonopodal solenomere tip, shape: sharp (0), DT = 0.7614; blunt (1), DT = 0.2386; HI = 0.9705.
36. ♂ Gonopodal third branch, presence: absent (0), DT = 0.9588; present (1), DT = 0.0412; HI = 0.7955.
37. ♂ Gonopodal acropodite, curvature: linear (0), DT = 0.5047; irregular circle (1), DT = 0.4457; smoothly continuous circle (2), DT = 0.0496; HI = 0.8211.
38. ♂ Gonopodal acropodite arc (viewed ventrally), curvature: linear, no arc (0), DT = 0.3167; ventrally (1), DT = 0.1149; laterally (2), DT = 0.4120; cephalically (3), DT = 0.1564; HI = 0.9508.
39. ♂ Gonopodal acropodite torsion, presence: absent (0), DT = 0.5186; present (1), DT = 0.4815; HI = 0.8958.
40. ♂ Gonopodal acropodite, bulk: thin (0), DT = 0.6440; bulky (1), DT = 0.3560; HI = 0.9949.
41. ♀ Cyphopodal receptacle, presence: present (0); absent (1).
42. ♀ Cyphopodal receptacle, size at its widest part: absent (0); shorter than prefemur length (1); subequal to prefemur length (2); wider than prefemur length (3).
43. ♀ Cyphopodal valves, symmetry: symmetric (0); asymmetric (1).
44. ♀ Cyphopodal valves, orientation: ventrally (0); anteroventrally (1); posteroventrally (2); twisted posterior (3).
45. ♀ Cyphopodal receptacle cuticle, surface: absent (0); smooth (1); sculptured (2).
46. ♂ Gnathochilarium lateral emargination, presence: present (0); absent (1).
47. ♂ Antennomere one distal cuticle, conformation*: cylindrical, not wrapped around cones (0); wrapped around cones (1).
48. ♂ Collum ridges, presence: present on anterolateral margin (0), DT = 0.9547; absent (1), DT = 0.0453; HI = 0.8773
49. ♂ Caudolateral corners, paranota I-X, shape: acute, projecting caudally (0), DT = 0.5552; rounded cephalically (1), DT = 0.4449; HI = 0.9769.
50. ♂ Caudolateral corners, paranota I-XIX, shape: acute, projecting caudally on all segments (0), DT = 0.4054; rounded cephalically on segments I-X only (1), DT = 0.4934; rounded cephalically on segments I-X, rounded throughout remaining segments (XI-XIX) (2), DT = 0.1010; HI = 0.9675.
51. ♂ Metatergal pores, metatergites IX+X, presence: present (0), DT = 0.9717; absent (1), DT = 0.0283; HI = 0.9943.
52. ♂ Metatergal linear bump-pores, presence: absent (0), DT = 0.8876; present (1), DT = 0.1124; HI = 0.8617.
53. ♂ Lateral wrinkles, tergites IX+X, presence: tightly wrinkled (0), DT = 0.4711; loosely wrinkled (1), DT = 0.5289; HI = 0.9861.
54. ♂ Longitudinal paranotal wrinkles, presence: present (0), DT = 0.1096; absent (1), DT = 0.8904; HI = 0.8028.
55. ♂ Repugnatorial glands, paranota IX+X, orientation: laterally (0), DT = 0.4781; dorsal (1), DT = 0.5219; HI = 0.9602.
56. ♂ Metatergal dorsal slits, presence: present (0), DT = 0.9785; absent (1), DT = 0.0215; HI = 0.8440.
57. ♂ Metatergal dorsal microsculpture, mesh shape: isodiametric (0), DT = 0.9518; anisodiametric (1), DT = 0.0482; HI = 0.6575.
58. ♂ Paranota-dorsum, segments IX+X, angle: 130 degrees (0), DT = 0.2811; 180 degrees (1), DT = 0.7189; HI = 0.8012.
59. ♂ Paranotal segments IX+X, width: thick (0), DT = 0.1064; thin (1), DT = 0.8936; HI = 0.8162.
60. ♂ Anterior dorsolateral paranotal disc, segments IX+X, concavity: puffed out (0), DT = 0.0837; flat (1), DT = 0.1644; scooped out (2), DT = 0.7519; HI = 0.9865.
61. ♂ Gonapophyses, shape: cylinder-shaped (0), DT = 0.5073; goblet-shaped (1), DT = 0.4927; HI = 0.9978.
62. ♂ Sternal knobs, 4th leg pair, presence: absent (0), DT = 0.7563; present (1), DT = 0.2437; HI = 0.9837.
63. ♂ Pleural process, segments IX+X, presence: absent (0), DT = 0.5474; present (1), DT = 0.4536; HI = 0.9764.
64. ♂ Sternal triangular spines, segments IX+X, presence: present (0), DT = 0.1960; absent (1), DT = 0.8041; HI = 0.9317.
65. ♂ Sternal median bulge, segments IX+X, presence: absent (0), DT = 0.8952; present (1), DT = 0.1048; HI = 0.3711.
66. ♂ Setae, sterna IX+X, presence: present (0), DT = 0.4987; absent (1), DT = 0.5013; HI = 0.9503.
67. ♂ Ventral excavation, sterna IX+X, presence: present (0), DT = 0.2917; absent (1), DT = 0.7083; HI = 0.9419.
68. ♂ Pregonopodal tarsal claws, shape: curved (0), DT = 0.3395; bisinuately curved (1), DT = 0.6605; twisted, spatulate (2), DT = 0.000; HI = 0.7845.
