# Supplementary material for: Is geography an accurate predictor of evolutionary history in the millipede family Xystodesmidae?
Source: PeerJ. 2017 Oct 12;5:e3854. doi: 10.7717/peerj.3854 (PMC5641431; doi:10.7717/peerj.3854)
Supplement: Appendix C — DNA amplification procedures for the six genes used in this study: COI; EF1a; tRNA-VAL, 12S and 16S (denoted as 12S–16S in text); and 28S. [file peerj-05-3854-s003.docx]

**Appendix C**

**Amplification Procedures**

For DNA amplification we used the following master mixes [volumes calculated for 6 samples plus a negative and a positive control (49 µL)] and thermocycler protocols.

*COI*: master mix - 303.8 µL water, 45 µL dNTP’s, 45 µL 10X buffer, 22.5 µL LCOI490 (forward primer, GGTCAACAAATCATAAAGATA, Folmer et al., 1994), 22.5 µL HCO2198 (reverse primer, TAAACTTCAGGGTGACCAAAAAATCA, Folmer et al., 1994) and 2.25 µL Taq polymerase (Takara Bio Inc., Japan); thermocycler protocol - start at 94 °C for 60 seconds, 5 cycles of denaturing (94 °C for 60 seconds), annealing (45 °C for 90 seconds), and extension (72 °C for 90 seconds), then 35 cycles of denaturing (94 °C for 60 seconds), annealing (50 °C for 90 seconds) and extension for 72 °C for 60 seconds) with a 5 minute final extension at 72 °C (Folmer et al. 1994, Hebert, Cywinska & Ball, 2003).

*EF1-a***:** master mix - 312.8 µL water, 36 µL dNTP’s, 45 µL 10X buffer, 22.5 µL DiploEF1aF (forward primer, GCCTGGGTTTTGGATAAACTTAAGGC, Sota and Tanabe, 2009), 22.5 µL DiploEF1aR3 (reverse, CCTCCAATCTTGTAAACGTC, Sota and Tanabe, 2009) and 2.25 µL Taq; thermocycler protocol - start at 94 °C for 120 seconds, 30 cycles of denaturing (94 °C for 20 seconds), annealing (50 °C for 20 seconds) and extension (72 °C for 45 seconds) with a 7 minute final extension at 72 °C (Sota and Tanabe, 2009).

*12S - 16S (3’ end)*: master mix - 290 µL water, 45 µL dNTP’s, 45 µL 10X buffer, 22.5 µL LR-J-APHE1 (forward primer, GTTTCACCTTCATACCAGC, Marek and Bond, 2006), 22.5 µL SR-N-145XXdip2 (reverse primer, GGACGTCAAGTCAAGGTGCAG, Marek and Bond, 2006), 13.5 µL DMSO and 2.25 µL Taq; thermocycler protocol - start at 95 °C for 120 seconds, then 29 cycles of denaturing (94 °C for 30 seconds), annealing (52 °C for 30 seconds) and extension (72 °C for 60 seconds) with a 2 minute final extension at 72 °C (Marek and Bond, 2006; 2007; Marek and Moore, 2015).

*12S - 16S (5’ end)*: master mix - 312.8 µL water, 45 µL dNTP’s, 45 µL 10X buffer, 11.25 µL LR-J-12887dip2 (forward primer, CCGGTCTGAACTCAGATCATGT, Marek and Bond, 2006), 11.25 µL SR-N-145XXdip2 (reverse primer, GGACGTCAAGTCAAGGTGCAG, Marek and Bond, 2006), 13.5 µL DMSO and 2.25 µL Taq; thermocycler protocol - Start at 95 °C for 120 seconds, then 29 cycles of denaturing (94 °C for 30 seconds), annealing (52 °C for 30 seconds) and extension (72 °C for 60 seconds) with a 2 minute final extension at 72 °C (Marek and Bond, 2006; 2007; Marek and Moore, 2015).

*28S*: master mix - 299.3 µL water, 36 µL dNTP’s, 45 µL 10X buffer, 22.5 µL D1 (forward primer, GGGAGGAAAAGAAACTAAC, Marek and Moore, 2015; Lavrov, Boore and Brown, 2002; Hillis and Dixon, 1991), 22.5 µL D3 (reverse primer, GCATAGTTCACCATCTTTC, Marek and Moore, 2015; Lavrov, Boore and Brown, 2002; Hillis and Dixon, 1991), 13.5 µL DMSO and 2.25 µL Taq; thermocycler protocol - start at 94 °C for 120 seconds, then 35 cycles of denaturing (94 °C for 20 seconds), annealing (52 °C for 20 seconds) and extension (72 °C for 70 seconds) with a 5 minute final extension at 72 °C (Marek and Moore, 2015; Maddison and Anderson, 2016).

**References**

Folmer O, Black M, Hoeh W, Lutz R, and Vrijenhoak R. 1994. DNA Primers for amplification of mitochondrial cytochrome *c* oxidase subunit I from diverse metazoan invertebrates. *Molecular marine biology and biotechnology* 3:294-299.

Hebert PDN, Cywinska A, and Ball SL. 2003. Biological identifications through DNA barcodes. *Proceedings of the Royal Society of London B: Biological Sciences* 270:313-321. DOI: 10.1098/rspb.2002.2218.

Hillis DM, and Dixon MT. 1991. Ribosomal DNA: molecular evolution and phylogenetic inference. *The Quarterly Review of Biology* 66:411-453. DOI: 10.1086/417338.

Lavrov DV, Boore JL, and Brown WM. 2002. Complete mtDNA sequences of two millipedes suggest a new model for mitochondrial gene rearrangements: duplication and nonrandom loss. *Molecular Biology and Evolution* 19:163-169. DOI: 10.1093/oxfordjournals.molbev.a004068.

Maddison DR, and Anderson R. 2016. Hidden species within the genus *Ocys* Stephens: the widespread species *O. harpaloides* (Audinet-Serville) and *O. tachysoides* (Antoine)(Coleoptera, Carabidae, Bembidiini). *Deutsche Entomologische Zeitschrift* 63:287-301. DOI: 10.3897/dez.63.10748.

Marek PE, and Bond JE. 2006. Phylogenetic systematics of the colorful, cyanide-producing millipedes of Appalachia (Polydesmida, Xystodesmidae, Apheloriini) using a total evidence Bayesian approach. *Molecular phylogenetics and evolution* 41:704-729. DOI: 10.1016/j.ympev.2006.05.043.

Marek PE, and Bond JE. 2007. A reassessment of apheloriine millipede phylogeny: additional taxa, Bayesian inference, and direct optimization (Polydesmida: Xystodesmidae). *Zootaxa* 1610:27-39.

Marek PE, and Moore W. 2015. Discovery of a glowing millipede in California and the gradual evolution of bioluminescence in Diplopoda. *Proceedings of the National Academy of Sciences* 112:6419-6424. DOI: 10.1073/pnas.1500014112.

Sota T, and Tanabe T. 2009. Multiple speciation events in an arthropod with divergent evolution in sexual morphology. *Proceedings of the Royal Society of London B: Biological Sciences*. DOI: 10.1098/rspb.2009.1822.
